# Genetic alterations of *TP53* and *OTX2* indicate increased risk of relapse in WNT medulloblastomas

**DOI:** 10.1007/s00401-022-02505-5

**Published:** 2022-10-01

**Authors:** Tobias Goschzik, Martin Mynarek, Evelyn Doerner, Alina Schenk, Isabel Spier, Monika Warmuth-Metz, Brigitte Bison, Denise Obrecht, Nina Struve, Rolf-Dieter Kortmann, Matthias Schmid, Stefan Aretz, Stefan Rutkowski, Torsten Pietsch

**Affiliations:** 1 Department of Neuropathology and DGNN Brain Tumor Reference Center, University of Bonn Medical Center, Venusberg-Campus 1, D-53127 Bonn, Germany; 2 Department of Pediatric Hematology/Oncology, University Clinics Hamburg-Eppendorf, Hamburg, Germany; 3 Mildred Scheel Cancer Career Center HaTriCS4, University Medical Center Hamburg-Eppendorf, Hamburg, Germany; 4 Institute of Medical Biometry, Informatics and Epidemiology, Medical Faculty, University of Bonn, Bonn, Germany; 5 Institute of Human Genetics and Center for Hereditary Tumor Syndromes, Medical Faculty, University of Bonn, Bonn, Germany; 6 Institute of Diagnostic and Interventional Neuroradiology, University Hospital Wuerzburg, Würzburg, Germany; 7 Department of Neuroradiology, University Hospital, Augsburg, Germany; 8 Department of Radiotherapy, University Medical Center Hamburg-Eppendorf, Hamburg, Germany; 9 Department of Radiation Oncology, University of Leipzig, Leipzig, Germany

**Keywords:** Medulloblastoma, WNT, OTX2, Prognosis

## Abstract

This study aimed to re-evaluate the prognostic impact of *TP53* mutations and to identify specific chromosomal aberrations as possible prognostic markers in WNT-activated medulloblastoma (WNT-MB). In a cohort of 191 patients with WNT-MBs, mutations in *CTNNB1*, *APC*, and *TP53* were analyzed by DNA sequencing. Chromosomal copy-number aberrations were assessed by molecular inversion probe technology (MIP), SNP6, or 850k methylation array hybridization. Prognostic impact was evaluated in 120 patients with follow-up data from the HIT2000 medulloblastoma trial or HIT registries. *CTNNB1* mutations were present in 92.2%, and *APC* mutations in 6.8% of samples. One *CTNNB1* wild-type tumor gained WNT activation due to homozygous *FBXW7* deletion. Monosomy 6 was present in 78.6%, and more frequent in children than adults. 16.1% of tumor samples showed *TP53* mutations, of those 60% with nuclear positivity for the p53 protein. Loss of heterozygosity at the *TP53* locus (chromosome 17p13.1) was found in 40.7% (11/27) of *TP53* mutant tumor samples and in 12.6% of *TP53* wild-type cases (13/103). Patients with tumors harboring *TP53* mutations showed significant worse progression-free survival (PFS; 5-year-PFS 68% versus 93%, *p* = 0.001), and were enriched for chromosomes 17p (*p* = 0.001), 10, and 13 losses. Gains of *OTX2* (14q22.3) occurred in 38.9% of samples and were associated with poor PFS and OS (5-year-PFS 72% versus 93%, *p* = 0.017 resp. 5-year-OS 83% versus 97%, *p* = 0.006). Multivariable Cox regression analysis for PFS/OS identified both genetic alterations as independent prognostic markers. Our data suggest that patients with WNT-MB carrying *TP53* mutations or *OTX2* gains (58.1%) are at higher risk of relapse. Eligibility of these patients for therapy de-escalation trials needs to be debated.

**Supplementary Information:**

The online version contains supplementary material available at 10.1007/s00401-022-02505-5.

## Introduction

Activation of the WNT signaling pathway is present in about 10% of all medulloblastomas (MB). These are now considered an independent MB entity with favorable outcome [11, 30]. WNT-pathway activation was first identified by immunohistochemical demonstration of nuclear *β*-catenin accumulation [11]. However, nuclear *β*-catenin accumulation alone is believed to be insufficient to detect all WNT-MBs as some cases lack detectable nuclear accumulation [12, 16, 26], and current neuropathological guidelines recommend the combination of different methods to identify WNT-MB.

In most WNT-MBs, pathway activation is a result of activating mutations in exon 3 of *CTNNB1*, leading to stabilization and subsequent nuclear accumulation of its product *β*-catenin [10, 13, 23, 47]. However, other members of the WNT signaling pathway like the tumor suppressor genes *APC*, *Axin1*, or *Axin2/Conductin* were shown to harbor mutations as alternative genetic alterations [9, 19, 22, 23], while only *CTNNB1* and *APC* mutations were found in a substantial fraction of WNT-MBs [33, 35, 38, 43, 44]. The favorable prognosis of WNT-MBs seems independent of the activating mutation [40, 43]. A substantial proportion of *APC*-mutant WNT-MB occur in the context of an *APC* germline mutation (either familial or de novo) [43]. In these cases, the MB is part of the tumor spectrum of familial adenomatous polyposis (FAP), and usually occurs as first manifestation of the disease before multiple colorectal adenomas are present.

In addition to *CTNNB1* and *APC* mutations that are considered the main drivers of WNT activation, several further recurrent mutations have been identified in WNT-MBs, including *DDX3X* (36%), *SMARCA4* (19%), and *TP53* (14%) [21]. Whereas in Sonic-Hedgehog (SHH)-MBs, *TP53* mutations were associated with very poor outcome, this was not the case in WNT-MBs [46]. Zhukova et al. showed a 5-year overall survival (OS) of 90% resp. 86% in discovery and validation cohorts in *TP53* mutant cases versus 97% and 94% in *TP53* wild-type WNT-MBs [46]. Lindsey et al. found *TP53* mutations in 3 of 15 *CTNNB1*-mutated MBs and all three patients showed long-term survival [29]. However, in a recent publication by Richardson et al., four of five relapsing WNT-MBs were described to carry *TP53* mutations [37].

Loss of chromosome 6 (monosomy 6) was found in the majority of WNT-MBs, with about 85% of pediatric WNT-MBs presenting monosomy 6 [6, 16, 20, 24, 30], but only 30–50% of adolescent and adult WNT-MB [5, 15]. Otherwise, WNT-MB samples present mainly stable genomes [21].

Given the very favorable prognosis with current standard therapy, patients with WNT-MB are enrolled in clinical studies aiming at a reduction of treatment intensity and hereby spare treatment-related side effects. Common to most of these trials is the reduction of radiotherapy dose to the whole brain and whole spine, either to 15 Gy (NCT01878617, St. Jude), or 18 Gy (NCT02724579, Children's Oncology Group, NCI, and NCT02066220, SIOP-Europe), which is very likely to reduce cognitive sequelae for the patients [31]. However, most patients with WNT-MB cannot be rescued when the disease recurs after standard therapy [32]. Therefore, it is of major importance to find biomarkers for identification of patients in which intensity of treatment should not be reduced, and of those patients qualifying for reduction of treatment intensity. In this large retrospective cohort, we aimed to re-evaluate the *TP53* status as prognostic factor in WNT-MBs, and to screen for chromosomal aberrations and other biomarkers as possible prognostic factors.

## Materials and methods

### Patients

Over a period of 20 years (2000–2020), 191 MB samples with confirmed WNT activation were identified. WNT-pathway activation was defined by the presence of at least two of the following features: nuclear positivity of β-catenin, mutation of a WNT-pathway component, e.g., *CTNNB1* (exon 3) or *APC*, and/or monosomy 6.

One-hundred and twenty patients participated in either the HIT-2000 trial (NCT00303810), the HIT-2000interim registry (NCT02238899), or the I-HIT-MED registry (NCT02417324), so detailed information on the treatment and follow-up was available (clinical cohort). The HIT2000 trial and both registries have been approved by the responsible ethics committees. Informed consent for exploratory research on tumor tissue had been given by the patients or their legal representatives.

### Histopathological and molecular analyses

According to the WHO classification 2016, the tumor samples were classified after conventional (HE and reticulin) and immunohistochemical stainings including markers for pathway activation [8, 34].

### Molecular inversion probe array (MIP)

A MIP array including 330,000 inversion probes (Version v2.0, Affymetrix, Santa Clara, CA, USA) was used as previously described to identify copy-number gains and losses in 108 samples [42]. The raw MIP data file was analyzed using the Nexus Copy Number 8.0 Discovery Edition software (BioDiscovery, El Segundo, CA, USA). BioDiscovery’s SNP-FASST2-Segmentation algorithm was used to make copy number and loss of heterozygosity calls. Further details are given in Online Resource Methods.

### Next-generation sequencing (NGS) and Sanger sequencing

Exon 3 of *CTNNB1* and exons 4–8 of *TP53* were directly analyzed by Sanger sequencing using specific primers in all resp. 186 samples. Furthermore, hotspot mutations at E17 in *AKT1* (exon 3), *AKT2* (exon 3), and *AKT3* (exon 2) were screened. Primer sequences are shown in Online Resource Table 1.

Forty WNT-MBs, including 13 *CTNNB1-*wild-type cases, were screened for *APC* mutations by one of two DNA-NGS panels as further described in Online Resource Methods and Online Resource Table 2.

### 450k/850k methylation bead arrays

Genome-wide methylation profiles were generated by hybridization to Illumina 450 k/850 k methylation bead arrays in 94 cases. The Heidelberg Brain Tumor classifier v11b4 was used to annotate the profiles to a methylation class [4, 18]; scores > 0.9 were considered as match. In 14 cases, copy-number information for numerical aberrations was retrieved from methylation bead arrays.

### Statistical analysis

Progression-free survival (PFS) and overall survival (OS) were defined as time from first tumor surgery to (a) first tumor progression or death of any cause (PFS) or (b) to death of any cause (OS). PFS and OS rates were estimated using the Kaplan–Meier method. Univariable survival rates were compared using the log-rank test. The most relevant risk factors were identified by a multivariable Cox regression analysis with variable selection independently for PFS and OS based on minimization of Akaike’s information criterion. For variable selection, each combination of the 12 covariates (therapy protocol, clinical risk, histology, *OTX2* status, *TP53* copy number, *TP53* mutation, chr6, chr10, chr13, chrX, WNT signaling activating mutation, and age at diagnosis) were considered (4096 different covariate combinations, including an intercept only model). Robustness of the results was investigated by repeatedly applying a subsampling approach independently for PFS and OS. Each of the 100 subsampling data sets consists of 80% of the original cohort whereby maintaining the ratio of events and non-events as in the complete cohort for PFS and OS, respectively. On each subsampling data set, Kaplan–Meier analyses were repeated and *p* value ranges for the log-rank test were obtained. Multivariable Cox regression analysis and variable selection procedure for the complete cohort were performed on each subsampling data set to investigate robustness of the derived model. The analyses have been performed in R version 4.1.1 with packages survival version 3.2–11 and survminer 0.4.9. All analyses were hypothesis-generating. *P *values were adjusted figurewise by the Bonferroni–Holm (BH) method to account for multiple testing. A (corrected) *p *value less than 0.05 was considered to be statistically significant.

## Results

### Cohort parameters

**Fig. 1:**
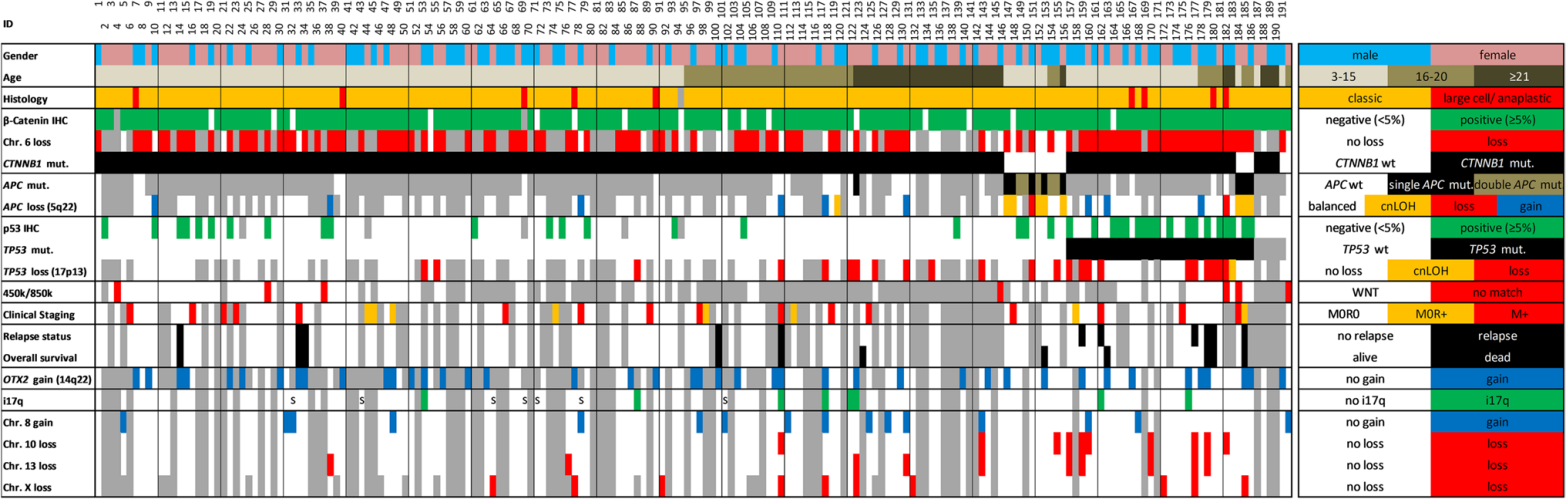
Clinical and molecular features of the WNT medulloblastoma cohort. A total of 191 patient samples with their mutational status (*CTNNB1*, *APC*, *TP53*) and most frequent focal and numerical chromosomal alterations are shown as well as nuclear accumulation of *β*-catenin and p53 protein. Survival data were available from 120 patients. Missing data are shown in gray. wt, wild-type; mut., mutation; IHC, immunohistochemistry; cnLOH, copy-neutral loss of heterozygosity; M0, no metastatic disease; M + , metastatic disease (M1-3); R0, gross-total resection (< 1.5 cm^2^); R + , subtotal resection; i17q, isochromosome 17q; Chr., chromosome; WCA, whole chromosomal aberrations; S, i17q and WCA data from SNP6 array instead of Molecular Inversion Probe array

In our cohort of 191 WNT-MBs, 125 patients were children (age 3–15 years), 36 were adolescents (16–20 years), and 30 were adults (≥ 21 years). The median age was 13 years. A female predominance was found (ratio 1.4:1 (Table 1, Online Resource Table 3). The majority of tumor samples had classic histology (*n* = 181; 94.8%); only nine cases presented with large cell/anaplastic (LC/A) histology (4.7%; Fig. 1, Online Resource Fig. 1 and Online Resource Table 4). Among the 120 patients with clinical data, 32 (26.7%) were clinically high-risk (HR); of these, 16 patients because of metastatic disease and the remaining because of large postoperative residual tumor or LC/A histology (eight patients each). Eighty-four patients received “standard-risk” medulloblastoma therapy with 23.4 Gy CSI with a boost to the posterior fossa or tumor bed of 54 Gy followed by maintenance chemotherapy or hyperfractionated radiotherapy with a CSI-dose of 36 Gy and boosts to posterior fossa and tumor bed according to the HIT-SIOP-PNET4 trial [27]. The remaining patients received “high-risk” medulloblastoma therapies, usually with 35.2 Gy CSI followed by a boost to the posterior fossa and maintenance chemotherapy according to the HIT’91 trial in the maintenance chemotherapy arm [17] or according to the MET-HIT2000-AB4 regimen (Table 1, Online Resource Table 5) [41].

**Table 1:** Cohort overview–demographical, molecular, and clinical characteristics

	Whole cohort *n* = 191	Clinical cohort with PFS/OS *n* = 120	Comparison *p* value*
Sex			
Male	79 (41.4%)	47 (39.2%)	*p* = 0.423
Female	112 (58.6%)	73 (60.8%)	
Male:female ratio	1: 1.42	1: 1.55	
Median age at diagnosis (range)	13 years (3–69)	11 years (3–31)	–
Histology			
Classic	181 (95.3%)	112 (93.3%)	*p* = 0.101
Large cell/anaplastic	9 (4.7%)	8 (6.7%)	
Not done	1	–	
WNT-activating mutation			
* CTNNB1*	176 (92.1%)	110 (91.7%)	*p* = 0.597
* APC*	13 (6.8%)	9 (7.5%)	
Other	2** (1.1%)	1^#^ (0.8%)	
*TP53* mutation	*n* = 186	*n* = 120	
Yes	30 (16.1%)	23 (19.2%)	*p* = 0.129
No	156 (83.9%)	97 (80.8%)	
*OTX2* status (chr. 14q22.3)	*n* = 108	*n* = 74	
Gain	42 (38.9%)	30 (40.5%)	*p* = 0.604
No gain	66 (61.1%)	44 (59.5%)	
Staging			
M0R0 (< 1.5 cm^2^)	–	96 (80%)	–
M0R +	–	8 (6.7%)	
M +	–	16 (13%)	
Therapy			
SR (23.4 Gy CSI + maintenance)	–	73 (61%)	–
SR (PNET4 HFRT)	–	11 (9.2%)	
HR (HIT91-E)	–	17 (14%)	
HR (MET-HIT-AB4)	–	15 (12%)	
HR (other schemes)	–	4 (3.3%)	

*SR* standard risk, *HR* high risk, *CSI* craniospinal irradiation, *HFRT* hyperfractionated radiotherapy

*Cohort with PFS/OS (n = 120) versus remaining cohort compared using Chi^2^-test

**1 *FBXW7* homozygous deletion; 1 × unknown (no *APC* sequencing)

^#^*FBXW7* homozygous deletion

**Fig. 2:**
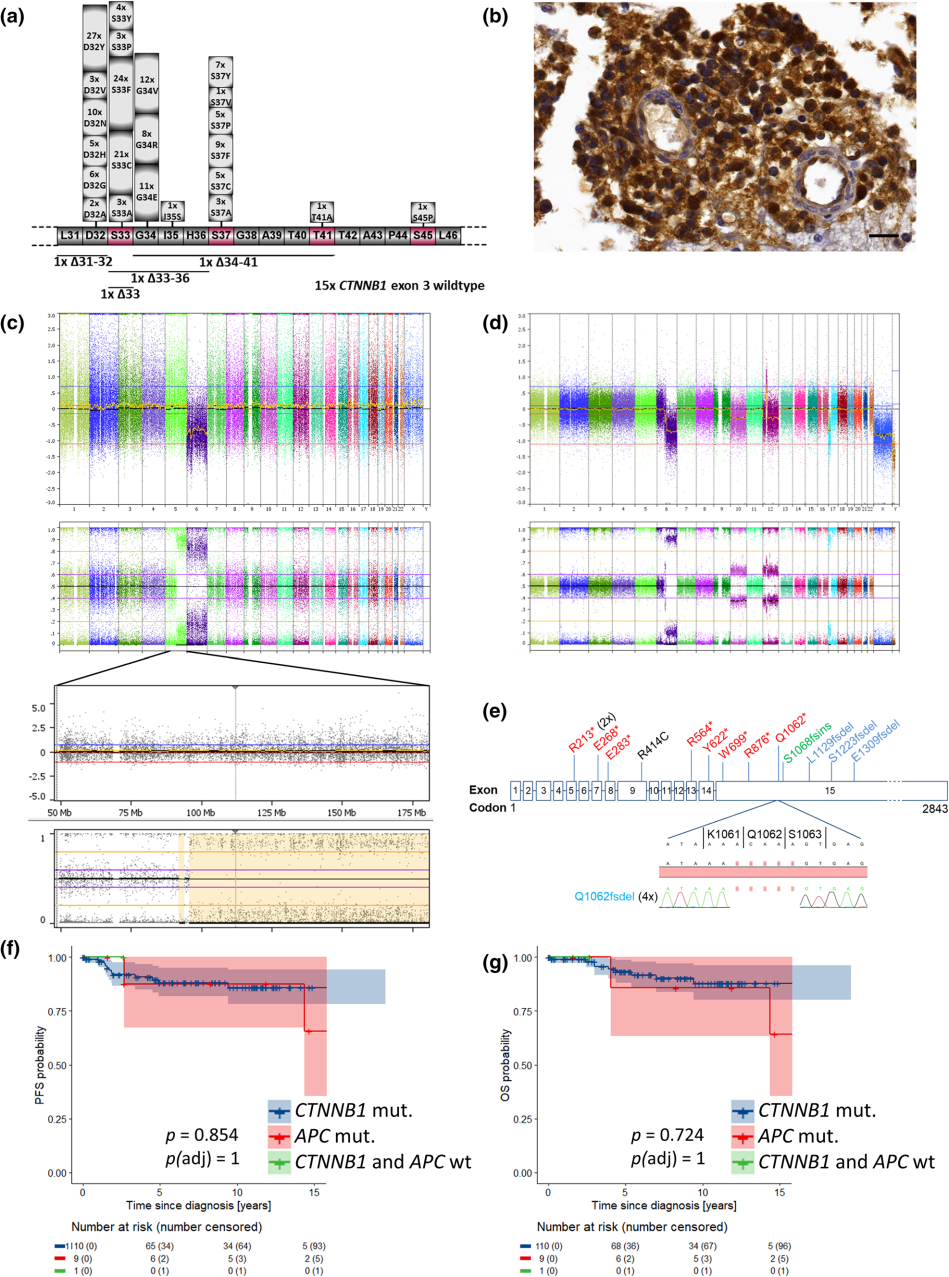
**a** Distribution of the 176 *CTNNB1* mutations in exon 3 at or adjacent to the 4 phosphorylation sites (purple color), including 4 short in-frame deletions. **b** Immunohistochemical staining of nuclear *β*-catenin accumulation in a WNT-MB (ID149) harboring two heterozygous *APC* mutations; normal cells with blue nuclei serve as internal control; scalebar = 20 µM. **c**, **d** Copy-number (upper) and allele ratio (lower) plots from molecular inversion probe array of a sibling pair (ID183 (**c**), ID154 (**d**)); the copy-neutral loss of heterozygosity within chromosome arm 5q in ID183 (**c**) is enlarged and the *APC* locus is highlighted by the gray vertical line; of note, ID154 (**d**) has additional mosaic losses of chromosomes 10, 12 and Y. **e** Distribution of *APC* mutations in 13 *CTNNB1* wild-type WNT-MBs; the homozygous R414C is the only missense mutation and likely benign according to the ACMG/AMP classification rules, but this case (ID151) has a second homozygous stop mutation (R283*) and a copy-neutral loss of heterozygosity, so that the R414C mutation will not be translated to protein and has no tumorigenic impact anyway. One *CTNNB1*-mutant WNT-MB (ID122) has a heterozygous R2226* *APC* mutation (not shown), which is likely pathogenic. **f**, **g** Kaplan–Meier progression-free (PFS) and overall survival (OS) plots of patients with *CTNNB1*-mutant versus *APC*-mutant WNT-MB; the green line shows ID191 without *CTNNB1* or *APC* mutation, but with a WNT-activating homozygous loss of *FBXW7*. The color-filled regions of the Kaplan–Meier plots indicate pointwise 95%-confidence intervals for the Kaplan–Meier estimate. The numbers at risk refer to the complete cohort. *p*(adj), adjusted *p* value after correction for multiple testing (Bonferroni–Holm)

Of the three major hallmarks of WNT-MBs, hotspot mutations in exon 3 of *CTNNB1* were most frequent (92.2%; 176 of 191), followed by nuclear accumulation of *β*-catenin protein (91.5% with ≥ 5% of nuclei; Fig. 2a, b). Monosomy 6 was present in 78.6% of analyzed cases (Fig. 1). The latter hallmark was more frequent in WNT-MBs of children (87.2%) compared with adolescents (70.4%) or adults (50%).

Of the 15 *CTNNB1*-wild-type tumors, 13 carried *APC* mutations. Of the remaining two tumor samples, one (ID191) had a homozygous deletion of the *FBXW7* gene located on chromosome 4q31.3 (Online Resource Fig. 2), whereas from the other sufficient DNA for *APC* sequencing was not available. However, this case qualified for the diagnosis of WNT-MB because of the presence of both nuclear *β*-catenin accumulation and monosomy 6. 450k/850k methylation array data was available in 94 cases, confirming the assignment to the WNT-MB group in 87 cases; in 7 cases, no matching score was reached (Fig. 1).

*TP53* mutations were found in 16.1% (30/186 cases; Table 1). Twenty six were missense mutations located in exons 5–8; only one was a missense variant in exon 4. Additionally, we found one splice site mutation and one frameshift deletion (both exon 6), and one case had a focal homozygous deletion of the *TP53* locus (chromosome 17p13.1; Fig. 4a, Online Resource Fig. 3). An accumulation of p53 protein in ≥ 5% of tumor cells was present in 20.4% of cases (38 of 186; Figs. 1, 4b). *TP53* mutations were found in 18 of 38 p53-positive tumor samples. The *TP53* locus showed loss of heterozygosity in 18.5% (24/130 cases), in some only present in a fraction of tumor cells (mosaicism). Of the 30 *TP53*-mutated cases, allele status was available from 27 samples, and 11 of these had a copy loss on chromosome 17p13.1. A significant predominance of male patients was found in cases with *TP53* mutations (18 of 30 (60%), *p* = 0.020, Chi-square test). *TP53* mutations occurred in all age groups (6–23 years) without a significant association with one of the age groups. *TP53* mutant and *TP53* wild-type cases did not cluster separately in UMAP analysis (Online Resource Fig. 4). LC/A histology was significantly enriched in *TP53*-mutated WNT-MBs (4 of 30 versus 5 of 156, *p* = 0.020, Chi-square test).

The E17K hotspot regions of *AKT1-3* were Sanger sequenced in 165 cases, and the E17K mutation was found 6 times each in *AKT1* and *AKT3* (12 of 165; 7.27%).

Next-generation panel sequencing (NGS panel II) was performed in 31 cases. Besides identification of *APC* variants and validation of *CTNNB1* and *TP53* mutations, recurrent mutations (*n* > 2) were found in *DDX3X* (*n* = 12, 38.7%), *SMARCA4* (*n* = 5), *KMT2D* (*n* = 5), *KMT2C* (*n* = 4), *PIK3CA* (*n* = 3), and *FBXW7* (*n* = 3; all mutations are shown in Online Resource Table 6).

### Chromosomal aberrations

Except for monosomy 6, most WNT-MBs presented stable genomes. Genome-wide copy-number profiles were available from 128 cases with a mean of 2.0 whole chromosomal aberrations (WCAs) per case. Most frequent WCAs besides chromosome 6 were gains of chromosomes 8 and 19 in 16 samples each (12.5%), and losses of chromosomes 10 and 13 in 10 samples each (7.8%; Fig. 4e, Online Resource Fig. 5). *TP53*-mutated tumors were enriched for monosomy 6 (28/28 cases versus 74/101 *TP53* wild-type tumors, *p* = 0.002, Chi-square test) and chromosome arm 17p loss (11/27 versus 13/88 *TP53* wild-type tumors, *p* = 0.001, Chi-square test; Online Resource Fig. 6). An isochromosome 17q (i17q) was found in 8 of 130 samples (6.2%). Genomic identification of significant targets in cancer (GISTIC) analysis revealed several significantly altered focal aberrations, most notably *CDK6* gain (chromosome 7q21.2), *OTX2* gain (14q22.3), and *AXIN2* gain (17q24.1; Online Resource Table 7 and Online Resource Fig. 7). Of note, *OTX2* gain (*n* = 42; Table 1, Fig. 4f and Online Resource Fig. 8) was the most frequent focal aberration occurring both in *TP53* mutant (8/27, 29.6%) and *TP53* wild-type (33/79, 41.8%, *p* = 0.263, chi-square test) tumors. Cases with *OTX2* gain versus balanced *OTX2* locus did not cluster separately in UMAP analysis (Online Resource Fig. 4).

### *APC* mutations

Of 13 *APC*-mutant cases, 8 had larger regions with copy-neutral loss of heterozygosity (cnLOH) spanning the *APC* locus up the telomeric region of chromosome 5q; one case had a focal loss at the *APC* locus (5q22.2). Eight of these nine cases with deletion carried a single mutation, and one carried a double mutation on the same allele (R283* and R414C) with allele variant fractions between 75 and 100%. The remaining four cases presented with two mutations with allele variant fractions of both mutations around 40–50% and 3 lacked LOH of the *APC* locus (1 × not done; Figs. 1, 2e). *TP53* mutations and *OTX2* gains both occurred at similar frequencies in *APC*-mutant WNT-MBs compared to *CTNNB1*-mutant tumors (*TP53* mutation: 3/13 versus 27/172, *p* = 0.486; *OTX2* gain: 4/12 versus 37/94, *p* = 0.686, Chi-square tests).

### Occurrence of *APC*-mutant WNT-MB in siblings

Our WNT-MB cohort contained one sibling pair with a female patient, who developed a classic MB at the age of 11 with metastatic spread at diagnosis (ID183). Her brother was 19 when the tumor was detected, and histology was also classic, but without metastatic disease (ID154). Both were *CTNNB1* wild-type, but *APC* sequencing revealed the same R213* mutation in both tumors, suggesting that it represented a germline mutation. The sister had an allele variant fraction of almost 100% and a large cnLOH on chromosome arm 5q, whereas the brother had a balanced *APC* locus, but an additional two base-pair frameshift deletion at amino acid L1129. Both *APC* mutations in this tumor had allele variant fractions of 40–50%.

Additionally, both siblings had no *OTX2* gains, but were affected by *TP53* alterations. The sister presented a monoallelic *TP53* mutation (R282P) without loss of the *TP53* locus, whereas the brother had a chromosome arm 17p loss, but wild-type *TP53* (Fig. 2c, d). Both siblings are alive without relapse or secondary tumor.

### Survival analyses

**Fig. 3:**
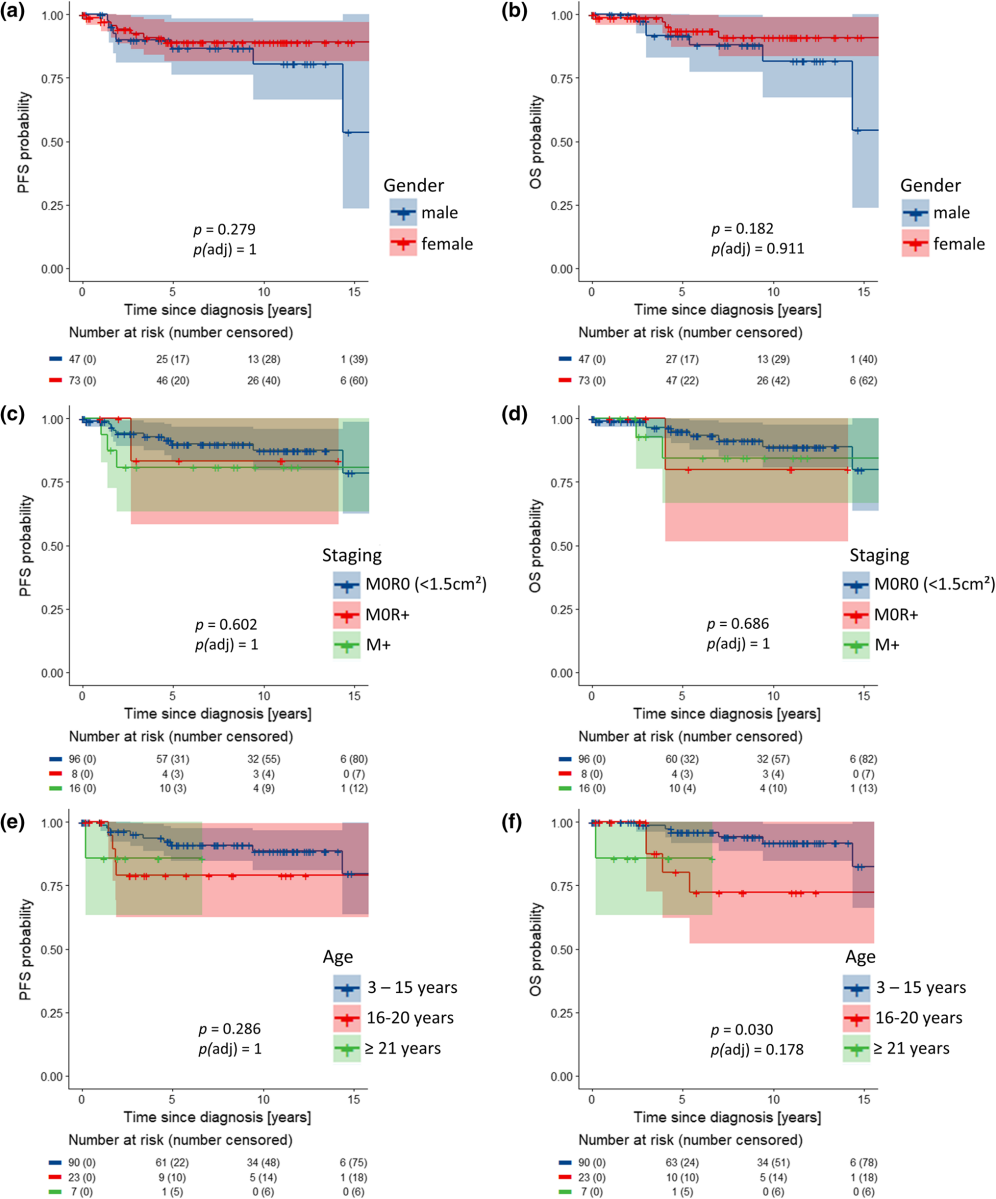
Kaplan–Meier progression-free (PFS) and overall survival (OS) plots for gender (**a**, **b**), staging with clinical high-risk factors “metastatic disease” (M +) and “subtotal resected tumor” (R + ; ≥ 1.5 cm^2^) versus cases with non-metastatic disease and gross-total resection (M0R0; **c**, **d**), and age (**e**, **f**). **e**, **f** Log-rank *p* values for individual PFS analyses are: blue versus red = 0.142; red versus green = 0.894; blue versus green = 0.284. Log-rank *p* values for individual OS analyses are: blue versus red = 0.020; red versus green = 0.856; blue versus green = 0.036

**Fig. 4:**
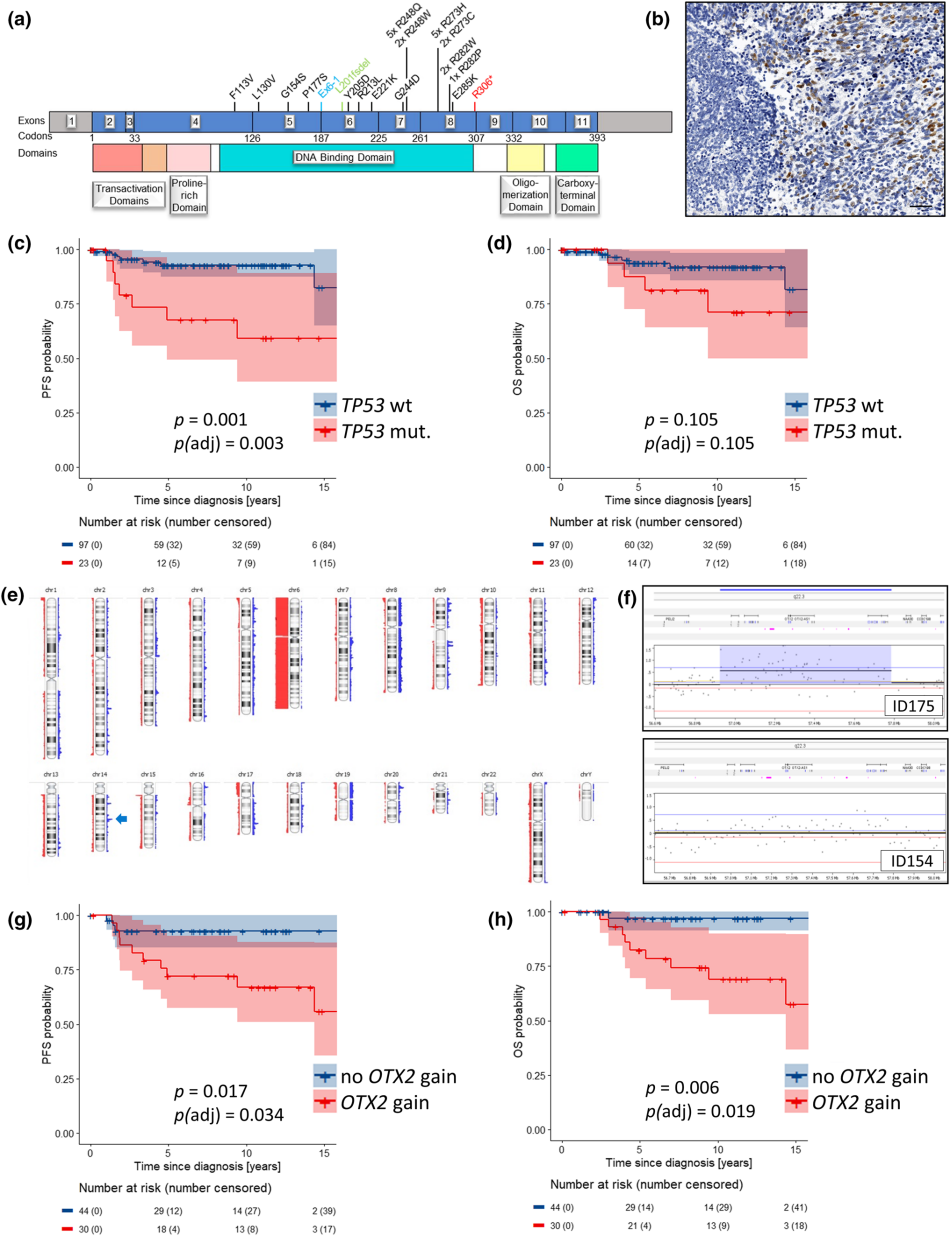
**a** Distribution of *TP53* mutations; colored mutations represent splice site mutation (blue), frameshift deletion (green), and nonsense mutation (red). **b** Immunohistochemical staining for p53 protein in a WNT-MB (ID184) with a heterozygous R248Q *TP53* mutation and balanced *TP53* locus; a strong nuclear staining pattern is visible focally, whereas some regions lack p53 staining (left side of the image); scalebar = 50 µM. **c**, **d** Kaplan–Meier progression-free (PFS) and overall survival (OS) plots of patients with *TP53* mutant versus *TP53* wild-type WNT-MB. **e** Summary plot of copy-number aberrations (*n* = 115 WNT-MBs) from molecular inversion probe array (*n* = 108) and SNP6 array (*n* = 7). The arrow indicates the *OTX2* locus on chromosome arm 14q. Blue bars, gains; red bars, losses. Thickness of bars indicates frequency of alterations. **f** Copy-number plots from molecular inversion probe arrays of a WNT-MB case with focal *OTX2* gain (upper part) and balanced *OTX2* (lower part) on chromosome arm 14q. **g**, **h** Kaplan–Meier PFS and OS plots of patients with *OTX2* gain versus no *OTX2* gain

**Fig. 5:**
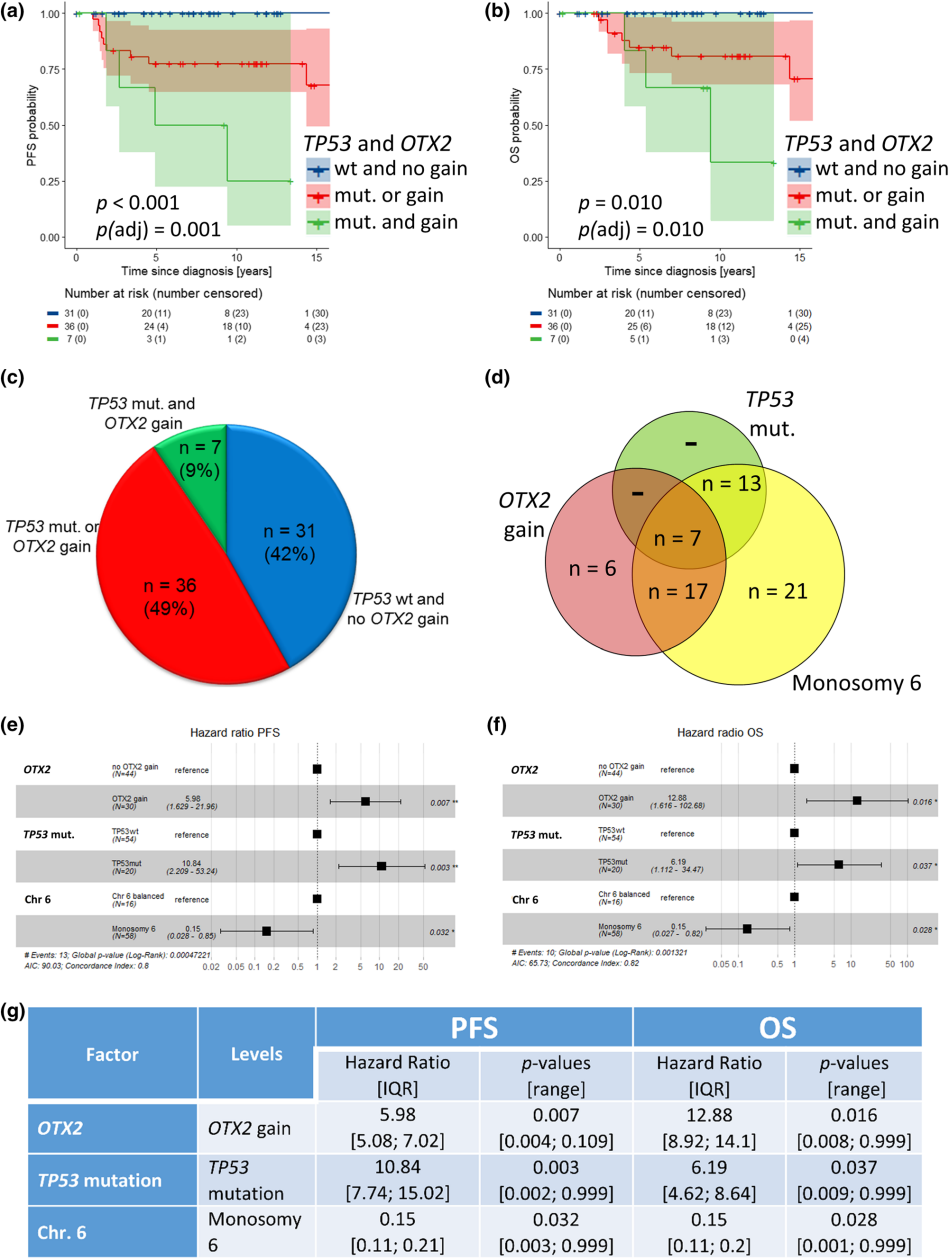
**a**, **b** Kaplan–Meier progression-free (PFS) and overall survival (OS) plots of patients with WNT-MBs without *TP53* mutation and *OTX2* gain (blue line), one of these two alterations (red line), or both alterations (green line). Log-rank *p* values for individual PFS analyses are: blue versus red = 0.010; red versus green = 0.050; blue versus green < 0.001. Log-rank *p* values for individual OS analyses are: blue versus red = 0.030; red versus green = 0.100; blue versus green < 0.001. **c** Pie chart showing frequency of identified risk factors in 74 WNT-MB cases with PFS/OS and genomic data from Molecular Inversion Probe array. **d** Venn diagram showing the distribution of *TP53* mutant, *OTX2* gained, and chromosome 6 lost (*n* = 64 with at least one of these alterations; *n* = 10 were wt/balanced for all 3 factors). **e**, **f** Results of the multivariable Cox regression analyses for PFS and OS; *, *p* < 0.05; **, *p* < 0.01. **g** Results of the subsampling approach for the multivariable Cox regression analysis with variable selection. Values in brackets refer to the IQR or the range for the subsampling approach, respectively. Values outside brackets refer to the model on the complete data (*n* = 74). PFS: Concordance Index [range]: 0.8 [0.76; 0.86], Median Rank: 8, Frequency Rank 1: 14%, Average AIC: 65.88; OS: Concordance Index [range]: 0.82 [0.75; 0.91], Median Rank: 3, Frequency Rank 1: 35%, Average AIC: 49.92

Staging and follow-up data from 120 patients were available with median follow-up of 7.4 years (range 0.1–19.6 years) in 109 patients alive at last follow-up. 5-year PFS was 88% [95% CI 82–95%] and 5-year OS was 93% [88–98%]. Univariable survival analysis did not show statistically significant associations of survival with gender and clinical HR stage (M + , residual disease; Fig. 3a–d), LC/A histology, nuclear *β*-catenin accumulation, i17q, *AKT1-3* mutations, *CDK6* gains, or monosomy 6 as well as clinical risk and therapy regimen (Online Resource Figs. 9–11). As in other studies, patients older than 16 had worse survival, reaching significance in OS (5-years OS 3–15 years 96% [92–100%] versus 16–20 years 80% [62–100%] versus ≥ 21 years 86% [63–100%], *p* = 0.030), but not in PFS (91% [85–98%] versus 79% [63–100%] versus 86% [63–100%], *p* = 0.286). After correction for multiple testing, the difference in age groups for OS did not reach significance anymore (*p*(adj) = 0.178; Fig. 3e, f). *APC*-mutated cases showed similar survival as *CTNNB1*-mutated cases (Fig. 2f, g). In contrast, *TP53* mutations were associated with lower PFS (68% [49–93%] versus 93% [87–99%], *p* = 0.001, *p*(adj) = 0.003) and OS (88% [83–100%] versus 94% [89–99%], *p* = 0.105, *p*(adj) = 0.105; Fig. 4c, d), and *TP53* copy loss also was associated with lower PFS (*p* = 0.004, *p*(adj) = 0.014), but not OS (Online Resource Fig. 12), as was loss of whole chromosome 10: PFS *p* < 0.001, *p*(adj) = 0.003; loss of chromosome 13: PFS *p* = 0.003, *p*(adj) = 0.017 (Online Resource Figs. 13–15). Importantly, gains of the *OTX2* locus were associated with significant worse PFS (5-year PFS 72% [57–91%] versus 93% [85–100%], *p* = 0.017, *p*(adj) = 0.034) and OS (5-year OS 82% [69–98%] versus 97% [91–100%], *p* = 0.006, *p*(adj) = 0.019; Fig. 4g, h), which was independent and additive of the effect of *TP53* mutations on univariable levels. No relapses or other events were observed among patients with tumors harboring neither *TP53* mutations nor *OTX2* gains (5-year PFS/OS 100%; Online Resource Table 8), whereas 5-year PFS/OS was 78% 65–93%/85% 73–98% in patients whose tumors either had a *TP53* mutation or an *OTX2* gain (*n* = 36, 48.7%) and was 50% [22–100]/83% [58–100%] among patients with both *TP53* mutation and *OTX2* gain (*n* = 7, *p* < 0.001, *p*(adj) = 0.001/*p* = 0.001, *p*(adj) = 0.010; Fig. 5a–c, Online Resource Fig. 16). The difference in patients whose tumors either had a *TP53* mutation or an *OTX2* gain for PFS seemed to be relatively stable under subsampling (*p* value ranging from 0 to 0.036 in 100 subsampling data sets; Online Resource Table 9).

This effect was retained in a multivariable Cox regression analysis for PFS and OS, where *OTX2* gain (HR 5.98 [1.63–22.0], *p* = 0.007/12.9 [1.6–103], *p* = 0.016), *TP53* mutations (HR 10.8 [2.2–53.2], *p* = 0.003/6.2 [1.1–34.5], *p* = 0.037), and monosomy 6 (HR 0.15 [0.03–0.85], *p* = 0.032/0.15 [0.027–0.82], *p* = 0.028) were identified as significant and independent prognostic markers (Fig. 5d–f). The model selection procedure applied to the 100 subsampling data sets revealed the model containing the three mentioned covariates to have the minimal average AIC as well as the minimal median rank when ranking models according to the AIC (PFS: average AIC = 65.88 OS: average AIC = 49.92; Fig. 5g and Online Resource Fig. 17).

## Discussion

Several ongoing clinical trials aim to improve quality of life for patients with WNT-MB by de-escalation of therapy, especially reduced radiation dosage. However, despite the excellent overall prognosis (of WNT-MB patients aged 15 years or younger), patients who relapse might not survive their disease. To prevent these patients from relapse, their upfront identification by suitable prognostic tumor markers is required. Currently, older age is considered the main risk factor for non-metastatic WNT-MB, but data of adolescent and adult WNT-MBs are scarce and results are not fully consistent [5, 7, 14, 15, 24, 25, 32, 36, 39, 44, 45].

### Limitation of clinical parameters as risk predictors

Data on survival of adult patients (≥ 21 years) in our cohort are sparse, but our data confirmed the worse outcome for adolescents (16–20 years). Conventional clinical parameters used for stratification into standard or high risk were not suitable as predictive markers in our WNT-MB cohort (Fig. 3c, d). Although data for WNT-MB with clinical high-risk features are rare, this observation is in line with published data [32, 41].

### *APC-*mutant MB shows similar outcomes as *CTNNB1*-mutated WNT-MBs

WNT-MBs with pathway-activating loss-of-function mutations in the *APC* gene had similar PFS/OS compared to *CTNNB1*-mutated cases, thus validating previous publications [40, 43]. This indicates that patients with *APC*-mutant WNT-MB might be included into therapy de-escalating clinical trials equally to *CTNNB1*-mutant WNT-MB in the absence of other risk factors.

In contrast to activating *CTNNB1* mutations, which almost always occur as somatic events in the tumor, pathogenic *APC* mutations frequently are germline events. These cause the cancer prediposition syndrome FAP, which is associated with a very high lifetime risk for colorectal cancer. As a consequence, patients with an *APC*-related MB should be offered genetic counseling and screened for *APC* germline mutations with the aim to offer surveillance for colorectal cancer [1].

### *TP53* mutation correlates to increased risk of recurrent disease

We identified *TP53* mutations as a robust marker for increased risk of relapse in WNT-MBs. This finding corresponds to the recent description of an over-representation of *TP53* mutant tumors in relapsed WNT-MB leading to the hypothesis of a prognostic impact of *TP53* mutations [37]. Zhukova et al. did not find an impact of *TP53* mutations on OS, but might have missed their prognostic relevance for PFS, as this was not analyzed [46]. In line with this publication, we also could not find a significant correlation of *TP53* mutations to worse OS. However, multivariable analysis also showed a prognostic impact of *TP53* mutations on OS in our cohort, although this finding was less significant than for PFS and has to be validated in future trial cohorts.

*TP53* loss also indicated significantly worse PFS, which was expected as loss at the *TP53* locus was highly associated with *TP53* mutations.

### Focal gain of the master regulator *OTX2* indicates poor prognosis

The master regulator gene *OTX2* encodes a transcription factor essential for the normal development of CNS tissues like brain, cerebellum, pineal gland, and eye [3]. Both in the developing cerebellum and cerebellar medulloblastoma, *OTX2* is highly expressed, and has been shown to possess oncogenic activity in non-WNT/non-SHH medulloblastomas [2]. Its obvious biological role in WNT-activated medulloblastoma has not been studied in detail so far. In this study, gains of *OTX2* were identified as negative and independent prognosticator. Indeed, all patients with tumors lacking a *TP53* mutation who died or had tumor relapse harbored an *OTX2* gain. Whereas IHC positivity of OTX2 protein is visible in almost all WNT- and non-WNT/non-SHH-MB, these mostly focal gains can only be found in a subset of WNT-activated tumors and similarly in non-WNT/non-SHH-MBs, but not in SHH-MBs. *OTX2* has been shown to be highly repressed in SHH-MB [28] and copy-number gains of *OTX2* have not been described in large series of SHH-MB [14, 15, 39]. In standard-risk non-WNT/non-SHH-MBs, *OTX2* gain was not associated with poor outcome [14]. As these gains are mostly focal, high-resolution technologies, such as MIP or gene-specific probes, should be used to detect this alteration. Of note, the copy-number information deduced from widely used DNA methylation might not be suitable to detect these *OTX2* gains.

### Multivariable analysis and proposal of a novel risk prediction model

In multivariable analysis, three genomic alterations were identified as independent indicators of outcome both PFS and OS, namely *TP53* mutation, *OTX2* gain, and monosomy 6. All relapsed patients could be identified by the first two alterations, so we suggest a risk prediction model based on these two markers. In this risk model including *TP53* mutation and/or *OTX2* gain, 58.1% of patients were identified having an increased risk for relapse of their disease after therapy according to the standard before the era of dose-reducing trials in WNT-MB. In this model, the remaining 41.9% of patients had WNT-MB without these genetic alterations and represented low-risk patients; no relapse occurred in these 31 patients (Fig. 5a–c). As our cohort of WNT-MB patients included patients with high-risk clinicopathological features, we also performed explorative subgroup analyses on “pure standard-risk” cohorts with patients who would had qualified for inclusion into the SIOP-PNET-5 MB (inclusion up to 16 years of age at diagnosis; *n* = 50) or into the SJMB12 and ACNS1422 studies (up to 22 years of age; *n* = 64). Of note, *OTX2* gain remained prognostic in the latter standard-risk cohort, although patient numbers are substantially reduced (Online Resource Fig. 18). As the nature of the study is retrospective and the patient cohort described here was not homogeneously treated in the framework of a single controlled trial, our proposed risk model needs validation in current or future therapeutic trials of WNT-MB patients to investigate the predictive power of the model. Upon confirmation, these genetic markers might allow to more precisely identify low-risk patients eligible for dose-sparing therapeutic strategies, while for the remaining patients, dose de-escalation should be avoided, but approaches aiming to increase survival might become of relevance.

## Supplementary Information

Below is the link to the electronic supplementary material.

Supplementary file1 (XLSX 134 KB)

Supplementary file2 (PDF 3012 KB)

## References

[1] Achatz MI, Porter CC, Brugieres L, Druker H, Frebourg T, Foulkes WD, Kratz CP, Kuiper RP, Hansford JR, Hernandez HS (2017). Cancer screening recommendations and clinical management of inherited gastrointestinal cancer syndromes in childhood. Clin Cancer Res.

[2] Adamson DC, Shi Q, Wortham M, Northcott PA, Di C, Duncan CG, Li J, McLendon RE, Bigner DD, Taylor MD (2010). OTX2 is critical for the maintenance and progression of Shh-independent medulloblastomas. Cancer Res.

[3] Beby F, Lamonerie T (2013). The homeobox gene Otx2 in development and disease. Exp Eye Res.

[4] Capper D, Jones DTW, Sill M, Hovestadt V, Schrimpf D, Sturm D, Koelsche C, Sahm F, Chavez L, Reuss DE (2018). DNA methylation-based classification of central nervous system tumours. Nature.

[5] Cavalli FMG, Remke M, Rampasek L, Peacock J, Shih DJH, Luu B, Garzia L, Torchia J, Nor C, Morrissy AS (2017). Intertumoral heterogeneity within medulloblastoma subgroups. Cancer Cell.

[6] Clifford SC, Lusher ME, Lindsey JC, Langdon JA, Gilbertson RJ, Straughton D, Ellison DW (2006). Wnt/Wingless pathway activation and chromosome 6 loss characterize a distinct molecular sub-group of medulloblastomas associated with a favorable prognosis. Cell Cycle.

[7] Coltin H, Sundaresan L, Smith KS, Skowron P, Massimi L, Eberhart CG, Schreck KC, Gupta N, Weiss WA, Tirapelli D (2021). Subgroup and subtype-specific outcomes in adult medulloblastoma. Acta Neuropathol.

[8] Crosier S, Hicks D, Schwalbe EC, Williamson D, Leigh Nicholson S, Smith A, Lindsey JC, Michalski A, Pizer B, Bailey S (2021). Advanced molecular pathology for rare tumours: a national feasibility study and model for centralised medulloblastoma diagnostics. Neuropathol Appl Neurobiol.

[9] Dahmen RP, Koch A, Denkhaus D, Tonn JC, Sorensen N, Berthold F, Behrens J, Birchmeier W, Wiestler OD, Pietsch T (2001). Deletions of AXIN1, a component of the WNT/wingless pathway, in sporadic medulloblastomas. Cancer Res.

[10] Eberhart CG, Tihan T, Burger PC (2000). Nuclear localization and mutation of beta-catenin in medulloblastomas. J Neuropathol Exp Neurol.

[11] Ellison DW, Onilude OE, Lindsey JC, Lusher ME, Weston CL, Taylor RE, Pearson AD, Clifford SC, United Kingdom Children's Cancer Study Group Brain Tumour C (2005). β-Catenin status predicts a favorable outcome in childhood medulloblastoma: the United Kingdom Children's Cancer Study Group Brain Tumour Committee. J Clin Oncol.

[12] Fattet S, Haberler C, Legoix P, Varlet P, Lellouch-Tubiana A, Lair S, Manie E, Raquin MA, Bours D, Carpentier S (2009). Beta-catenin status in paediatric medulloblastomas: correlation of immunohistochemical expression with mutational status, genetic profiles, and clinical characteristics. J Pathol.

[13] Giles RH, van Es JH, Clevers H (2003). Caught up in a Wnt storm: Wnt signaling in cancer. Biochim Biophys Acta.

[14] Goschzik T, Schwalbe EC, Hicks D, Smith A, Zur Muehlen A, Figarella-Branger D, Doz F, Rutkowski S, Lannering B, Pietsch T (2018). Prognostic effect of whole chromosomal aberration signatures in standard-risk, non-WNT/non-SHH medulloblastoma: a retrospective, molecular analysis of the HIT-SIOP PNET 4 trial. Lancet Oncol.

[15] Goschzik T, Zur Muehlen A, Doerner E, Waha A, Friedrich C, Hau P, Pietsch T (2021). Medulloblastoma in adults: cytogenetic phenotypes identify prognostic subgroups. J Neuropathol Exp Neurol.

[16] Goschzik T, Zur Muhlen A, Kristiansen G, Haberler C, Stefanits H, Friedrich C, von Hoff K, Rutkowski S, Pfister SM, Pietsch T (2015). Molecular stratification of medulloblastoma: comparison of histological and genetic methods to detect Wnt activated tumours. Neuropathol Appl Neurobiol.

[17] Hoff KV, Hinkes B, Gerber NU, Deinlein F, Mittler U, Urban C, Benesch M, Warmuth-Metz M, Soerensen N, Zwiener I (2009). Long-term outcome and clinical prognostic factors in children with medulloblastoma treated in the prospective randomised multicentre trial HIT'91. Eur J Cancer.

[18] Hovestadt V, Remke M, Kool M, Pietsch T, Northcott PA, Fischer R, Cavalli FM, Ramaswamy V, Zapatka M, Reifenberger G (2013). Robust molecular subgrouping and copy-number profiling of medulloblastoma from small amounts of archival tumour material using high-density DNA methylation arrays. Acta Neuropathol.

[19] Huang H, Mahler-Araujo BM, Sankila A, Chimelli L, Yonekawa Y, Kleihues P, Ohgaki H (2000). APC mutations in sporadic medulloblastomas. Am J Pathol.

[20] Jones DT, Jager N, Kool M, Zichner T, Hutter B, Sultan M, Cho YJ, Pugh TJ, Hovestadt V, Stutz AM (2012). Dissecting the genomic complexity underlying medulloblastoma. Nature.

[21] Juraschka K, Taylor MD (2019). Medulloblastoma in the age of molecular subgroups: a review. J Neurosurg Pediatr.

[22] Koch A, Hrychyk A, Hartmann W, Waha A, Mikeska T, Waha A, Schuller U, Sorensen N, Berthold F, Goodyer CG (2007). Mutations of the Wnt antagonist AXIN2 (Conductin) result in TCF-dependent transcription in medulloblastomas. Int J Cancer.

[23] Koch A, Waha A, Tonn JC, Sorensen N, Berthold F, Wolter M, Reifenberger J, Hartmann W, Friedl W, Reifenberger G (2001). Somatic mutations of WNT/wingless signaling pathway components in primitive neuroectodermal tumors. Int J Cancer.

[24] Kool M, Korshunov A, Remke M, Jones DT, Schlanstein M, Northcott PA, Cho YJ, Koster J, Schouten-van Meeteren A, van Vuurden D (2012). Molecular subgroups of medulloblastoma: an international meta-analysis of transcriptome, genetic aberrations, and clinical data of WNT, SHH, Group 3, and Group 4 medulloblastomas. Acta Neuropathol.

[25] Korshunov A, Remke M, Werft W, Benner A, Ryzhova M, Witt H, Sturm D, Wittmann A, Schottler A, Felsberg J (2010). Adult and pediatric medulloblastomas are genetically distinct and require different algorithms for molecular risk stratification. J Clin Oncol.

[26] Korshunov A, Sahm F, Zheludkova O, Golanov A, Stichel D, Schrimpf D, Ryzhova M, Potapov A, Habel A, Meyer J (2019). DNA methylation profiling is a method of choice for molecular verification of pediatric WNT-activated medulloblastomas. Neuro Oncol.

[27] Lannering B, Rutkowski S, Doz F, Pizer B, Gustafsson G, Navajas A, Massimino M, Reddingius R, Benesch M, Carrie C (2012). Hyperfractionated versus conventional radiotherapy followed by chemotherapy in standard-risk medulloblastoma: results from the randomized multicenter HIT-SIOP PNET 4 trial. J Clin Oncol.

[28] Lin CY, Erkek S, Tong Y, Yin L, Federation AJ, Zapatka M, Haldipur P, Kawauchi D, Risch T, Warnatz HJ (2016). Active medulloblastoma enhancers reveal subgroup-specific cellular origins. Nature.

[29] Lindsey JC, Hill RM, Megahed H, Lusher ME, Schwalbe EC, Cole M, Hogg TL, Gilbertson RJ, Ellison DW, Bailey S (2011). TP53 mutations in favorable-risk Wnt/Wingless-subtype medulloblastomas. J Clin Oncol.

[30] Louis DN, Ohgaki H, Wiestler OD, Cavenee WK (2016) WHO Classification of Tumours of the Central Nervous System. International Agency for Research on Cancer, Lyon

[31] Michalski JM, Janss AJ, Vezina LG, Smith KS, Billups CA, Burger PC, Embry LM, Cullen PL, Hardy KK, Pomeroy SL (2021). Children's Oncology Group Phase III trial of reduced-dose and reduced-volume radiotherapy with chemotherapy for newly diagnosed average-risk medulloblastoma. J Clin Oncol.

[32] Nobre L, Zapotocky M, Khan S, Fukuoka K, Fonseca A, McKeown T, Sumerauer D, Vicha A, Grajkowska WA, Trubicka J (2020). Pattern of relapse and treatment response in WNT-activated medulloblastoma. Cell Rep Med.

[33] Northcott PA, Buchhalter I, Morrissy AS, Hovestadt V, Weischenfeldt J, Ehrenberger T, Grobner S, Segura-Wang M, Zichner T, Rudneva VA (2017). The whole-genome landscape of medulloblastoma subtypes. Nature.

[34] Pietsch T, Haberler C (2016). Update on the integrated histopathological and genetic classification of medulloblastoma—a practical diagnostic guideline. Clin Neuropathol.

[35] Pugh TJ, Weeraratne SD, Archer TC, Pomeranz Krummel DA, Auclair D, Bochicchio J, Carneiro MO, Carter SL, Cibulskis K, Erlich RL (2012). Medulloblastoma exome sequencing uncovers subtype-specific somatic mutations. Nature.

[36] Remke M, Hielscher T, Northcott PA, Witt H, Ryzhova M, Wittmann A, Benner A, von Deimling A, Scheurlen W, Perry A (2011). Adult medulloblastoma comprises three major molecular variants. J Clin Oncol.

[37] Richardson S, Hill RM, Kui C, Lindsey JC, Grabovksa Y, Keeling C, Pease L, Bashton M, Crosier S, Vinci M (2022). Emergence and maintenance of actionable genetic drivers at medulloblastoma relapse. Neuro Oncol.

[38] Robinson G, Parker M, Kranenburg TA, Lu C, Chen X, Ding L, Phoenix TN, Hedlund E, Wei L, Zhu X (2012). Novel mutations target distinct subgroups of medulloblastoma. Nature.

[39] Shih DJ, Northcott PA, Remke M, Korshunov A, Ramaswamy V, Kool M, Luu B, Yao Y, Wang X, Dubuc AM (2014). Cytogenetic prognostication within medulloblastoma subgroups. J Clin Oncol.

[40] Surun A, Varlet P, Brugieres L, Lacour B, Faure-Conter C, Leblond P, Bertozzi-Salomon AI, Berger C, Andre N, Sariban E (2020). Medulloblastomas associated with an APC germline pathogenic variant share the good prognosis of CTNNB1-mutated medulloblastomas. Neuro Oncol.

[41] von Bueren AO, Kortmann RD, von Hoff K, Friedrich C, Mynarek M, Muller K, Goschzik T, Zur Muhlen A, Gerber N, Warmuth-Metz M (2016). Treatment of children and adolescents with metastatic medulloblastoma and prognostic relevance of clinical and biologic parameters. J Clin Oncol.

[42] Wang Y, Cottman M, Schiffman JD (2012). Molecular inversion probes: a novel microarray technology and its application in cancer research. Cancer Genet.

[43] Waszak SM, Northcott PA, Buchhalter I, Robinson GW, Sutter C, Groebner S, Grund KB, Brugieres L, Jones DTW, Pajtler KW (2018). Spectrum and prevalence of genetic predisposition in medulloblastoma: a retrospective genetic study and prospective validation in a clinical trial cohort. Lancet Oncol.

[44] Wong GC, Li KK, Wang WW, Liu AP, Huang QJ, Chan AK, Poon MF, Chung NY, Wong QH, Chen H (2020). Clinical and mutational profiles of adult medulloblastoma groups. Acta Neuropathol Commun.

[45] Zhao F, Ohgaki H, Xu L, Giangaspero F, Li C, Li P, Yang Z, Wang B, Wang X, Wang Z (2016). Molecular subgroups of adult medulloblastoma: a long-term single-institution study. Neuro Oncol.

[46] Zhukova N, Ramaswamy V, Remke M, Pfaff E, Shih DJ, Martin DC, Castelo-Branco P, Baskin B, Ray PN, Bouffet E (2013). Subgroup-specific prognostic implications of TP53 mutation in medulloblastoma. J Clin Oncol.

[47] Zurawel RH, Chiappa SA, Allen C, Raffel C (1998). Sporadic medulloblastomas contain oncogenic beta-catenin mutations. Cancer Res.

