# Supplementary material for: Genetic alterations of TP53 and OTX2 indicate increased risk of relapse in WNT medulloblastomas
Source: Acta Neuropathol. 2022 Oct 1;144(6):1143–56. doi: 10.1007/s00401-022-02505-5 (PMC9637613; doi:10.1007/s00401-022-02505-5)
Supplement: Supplementary file 2 — Supplementary file2 (PDF 3012 KB) [file 401_2022_2505_MOESM2_ESM.pdf]

Genetic alterations of *TP53* and *OTX2* indicate increased risk of relapse in WNT medulloblastomas

Online Resource Methods & Figures

Acta Neuropathologica

Tobias Goschzik, Martin Mynarek, Evelyn Doerner, Alina Schenk, Isabel Spier, Monika Warmuth-Metz, Brigitte Bison, Denise Obrecht, Nina Struve, Rolf-Dieter Kortmann, Matthias Schmid, Stefan Aretz, Stefan Rutkowski & Torsten Pietsch

Corresponding author
Torsten Pietsch, MD, Institute of Neuropathology, University of Bonn Medical Center, Venusberg-Campus 1, D-53127 Bonn, Germany, Phone +492282876602, Fax +4922828714331, Email t.pietsch@uni-bonn.de

CONTENTS

Methods — Page

1. Molecular Inversion Probe array 2
2. Next-Generation Sequencing 2

Figures

1. LC/A histology 4
2. Homozygous deletion of *FBXW7* (ID191) 4
3. Homozygous deletion of *TP53* (ID158) 5
4. UMAP plots 6
5. Comparison plot: Female versus male 7
6. Comparison plots: *TP53* mut. versus *TP53* wt 8
7. GISTIC analysis 9
8. Extent of *OTX2* gains (MIP array) 10
9. Kaplan-Meier plots: Histology – β-catenin IHC - i17q 11
10. Kaplan-Meier plots: *AKT1-3* E17K mut. - *CDK6* gain - Monosomy 6 12
11. Kaplan-Meier plots: Clinical risk – Therapy 13
12. Kaplan-Meier plots: *TP53* loss – p53 IHC 14
13. Kaplan-Meier plots: Chr. 10 loss – Chr. 13 loss – Chr. X loss 15
14. Kaplan-Meier plots: Chr. 8 gain – Chr. 19 gain 16
15. Comparison plots: Relapse and Death 17
16. Kaplan-Meier plots: *TP53* mut. / *OTX2* gain 18
17. Kaplan-Meier plots: *TP53 / OTX 2* after subsampling 19
18. Kaplan-Meier plots: „pure“ SR cohorts 20-23

Online Resource Methods

Molecular Inversion Probe array

When at least 90% of the probe signals of a chromosome were above/below the defined thresholds, these chromosomes were counted as whole chromosome gains/losses. For acrocentric chromosomes (chr. 13, 14, 15, 21, and 22) only q-arms were analyzed. Sex chromosomes were excluded from the determination of the numerical extent of WCAs.[1] Genomic Identification of Significant Targets in Cancer (GISTIC) analysis was also used to identify and distinguish significant focal chromosomal aberrations from random background (*p*-level: 0.005). [2]

In few cases, only SNP6 data were available (from fresh-frozen material). These were only used for determination of numerical aberrations, but not focal gains/losses (*OTX2*, *CDK6*, GISTIC).

Next-Generation Sequencing (NGS) and Sanger sequencing

Sanger sequencing of exons 4-8 from *TP53* was performed in 186 cases; in all but 10 cases also exon 9 was included. Five cases were also analyzed for mutations in exon 10.

In 9 *CTNNB1*-wild type cases, a mutation analysis of the *APC* gene was performed using tumor-derived DNA as previously described.[3] Briefly, NGS targeted mutation screening was performed using a customized TruSight™ Cancer Sequencing kit (Illumina, San Diego, CA, USA) which encompassed 143 relevant genes associated with hereditary tumor syndromes (Gene-panel I in Supplemental Table 1). Library preparation, target enrichment, and high-throughput sequencing were performed according to the manufacturer's protocol. All samples were sequenced on an Illumina MiSeq sequencer. Alignment was performed using the software of the Illumina MiSeq sequencer and data analysis with the SeqPilot software (JSI Medical Systems) based on hg19.

Further 31 samples (including 4 other *CTNNB1*-wildtype cases) were assessed using an Illumina DNA Prep for Enrichment (Nextera Flex) NGS panel (Illumina, San Diego, CA, USA). Tumor-derived DNA Libraries were generated from 100-400ng FFPE DNA with ~9000 custom enrichment probes. These probes covered the coding regions and exon-intron boundaries of 87 genes or their mutational hotspots (if known) frequently mutated in MB (gene-panel II in Supplemental Table 1). Purified and normalized libraries were pooled and subsequently sequenced on a MiSeq System (Illumina) with reagent kit v2 (TSCA).

(cont. on next page)

Next-Generation Sequencing (NGS) and Sanger sequencing (cont.)
Alignment of reads, variant calling, and annotation was performed with BWA using the Illumina MiSeq Reporter and Variant Studio v3.0 (Illumina) data analysis software. Filtering and selection of variants was done manually using the Illumina Variant Studio v3.0 (Illumina) software.

The *APC* mutation in ID147 was only found by Sanger sequencing of a region around Q1062 in exon 15. Further NGS analysis was not done in this case.

References

1. Goschzik T, Schwalbe EC, Hicks D, Smith A, Zur Muehlen A, Figarella-Branger D et al (2018) Prognostic effect of whole chromosomal aberration signatures in standard-risk, non-WNT/non-SHH medulloblastoma: a retrospective, molecular analysis of the HIT-SIOP PNET 4 trial. Lancet Oncol 19(12):1602-1616. doi: 10.1016/S1470-2045(18)30532-1
2. Beroukhim R, Getz G, Nghiemphu L, Barretina J, Hsueh T, Linhart D et al (2007) Assessing the significance of chromosomal aberrations in cancer: methodology and application to glioma. Proc Natl Acad Sci U S A 104(50):20007-12. doi: 10.1073/pnas.0710052104
3. Henn J, Spier I, Adam RS, Holzapfel S, Uhlhaas S, Kayser K et al (2019) Diagnostic yield and clinical utility of a comprehensive gene panel for hereditary tumor syndromes. Hered Cancer Clin Pract 17:5. doi: 10.1186/s13053-018-0102-4

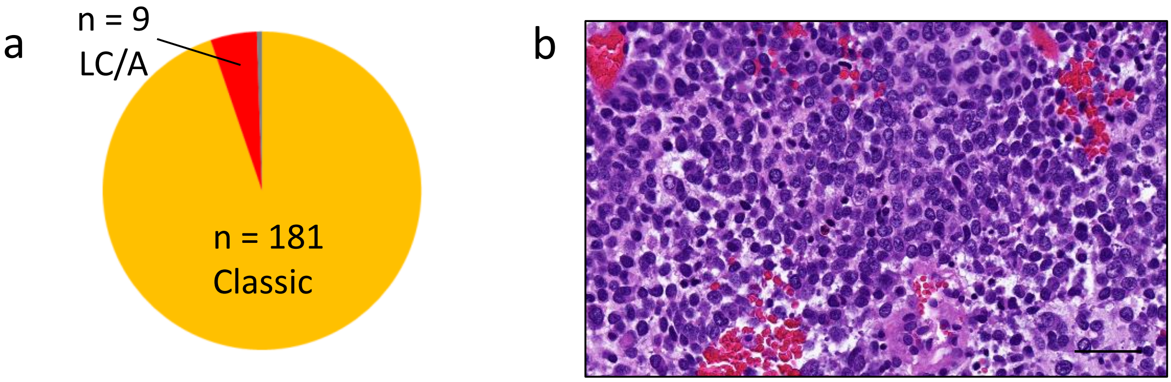

Online Resource Fig. 1: LC/A histology

(a) LC/A histology was found in 9 cases (red); 181 samples showed classic histology (yellow). In one case histological evaluation was not available (grey). **(b)** Representative example of a large-cell medulloblastoma (ID168) with prominent nucleoli; scalebar = 50 µM.

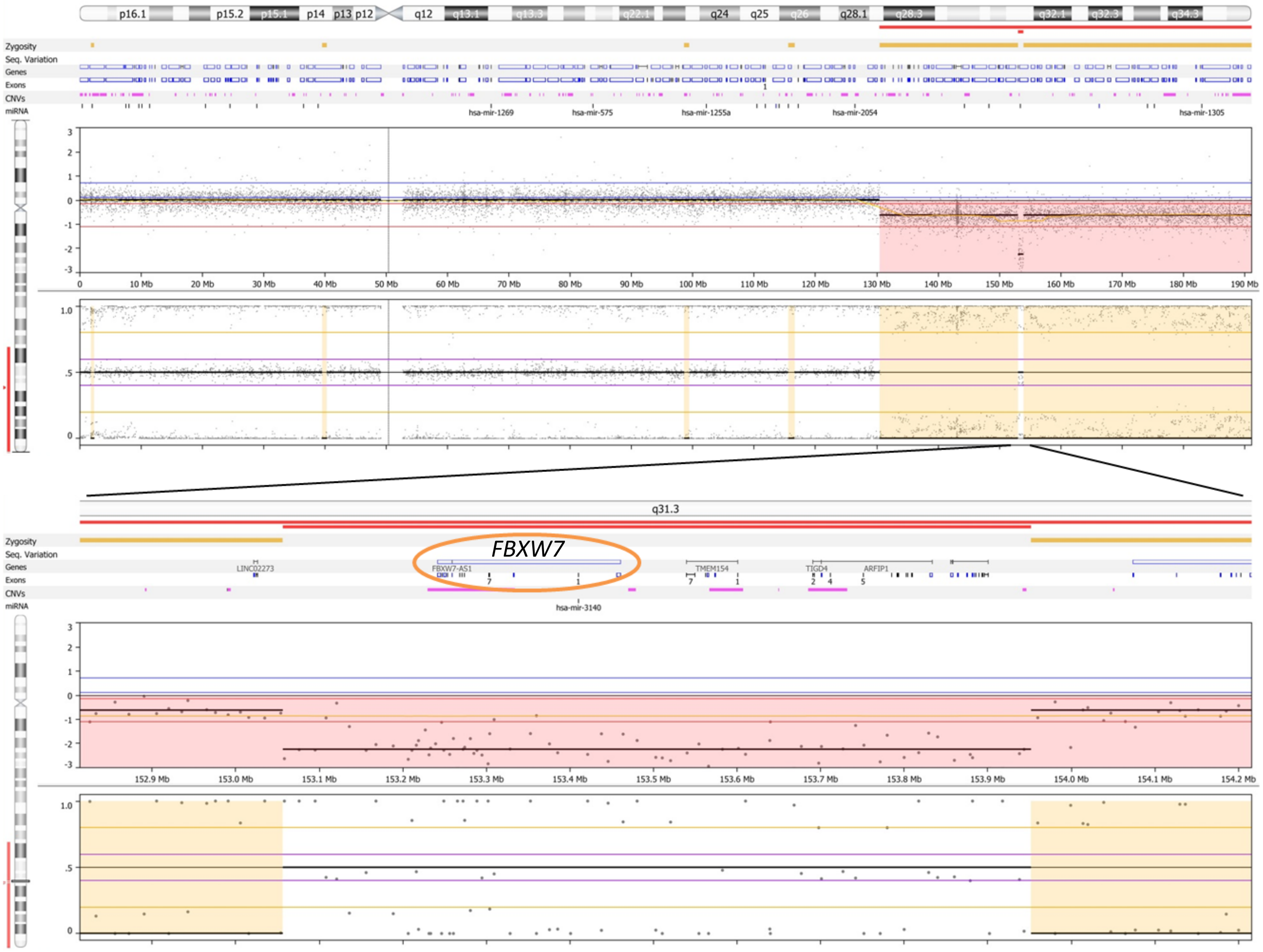

Online Resource Fig. 2: Homozygous deletion of the *FBXW7* gene locus on chromosome 4q31.3 (ID191)

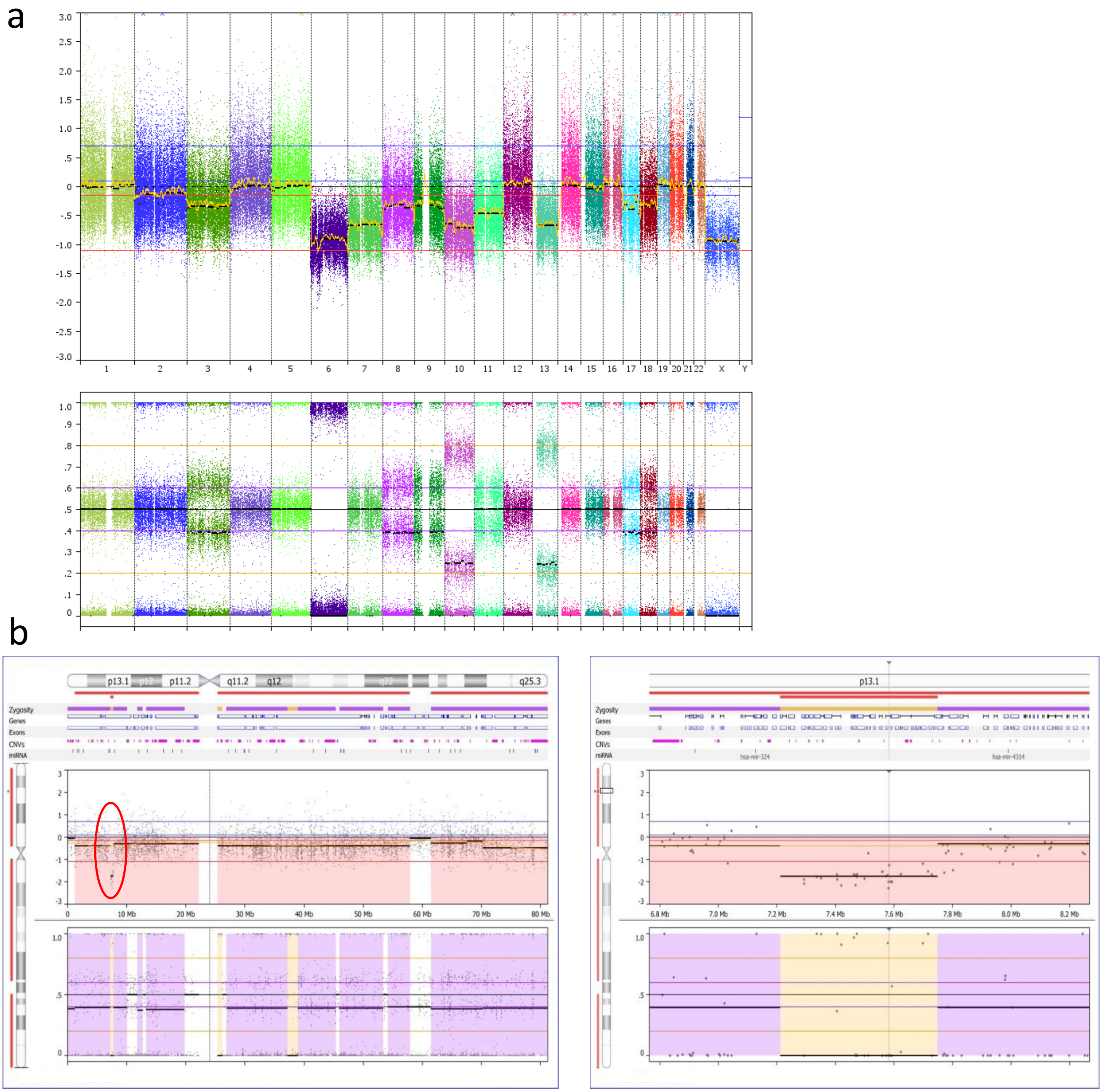

Online Resource Fig. 3: Whole genome view of a WNT-medulloblastoma (ID158) with a focal homozygous deletion of the *TP53* gene locus

(a) Whole genome view with copy number plot (upper part) and allele ratio (lower part). Several whole chromosomal losses are present, but mostly with different states of mosaicism. Only chromosome 6 is lost in all tumor cells (monosomy 6) and looks identical like the X chromosome of this male patient.

(b) Whole chromosome 17 (left) and enlarged *TP53* locus on chromosomal region 17p13.1 (right). The grey line in the right plot indicates the extact position of the *TP53* gene.

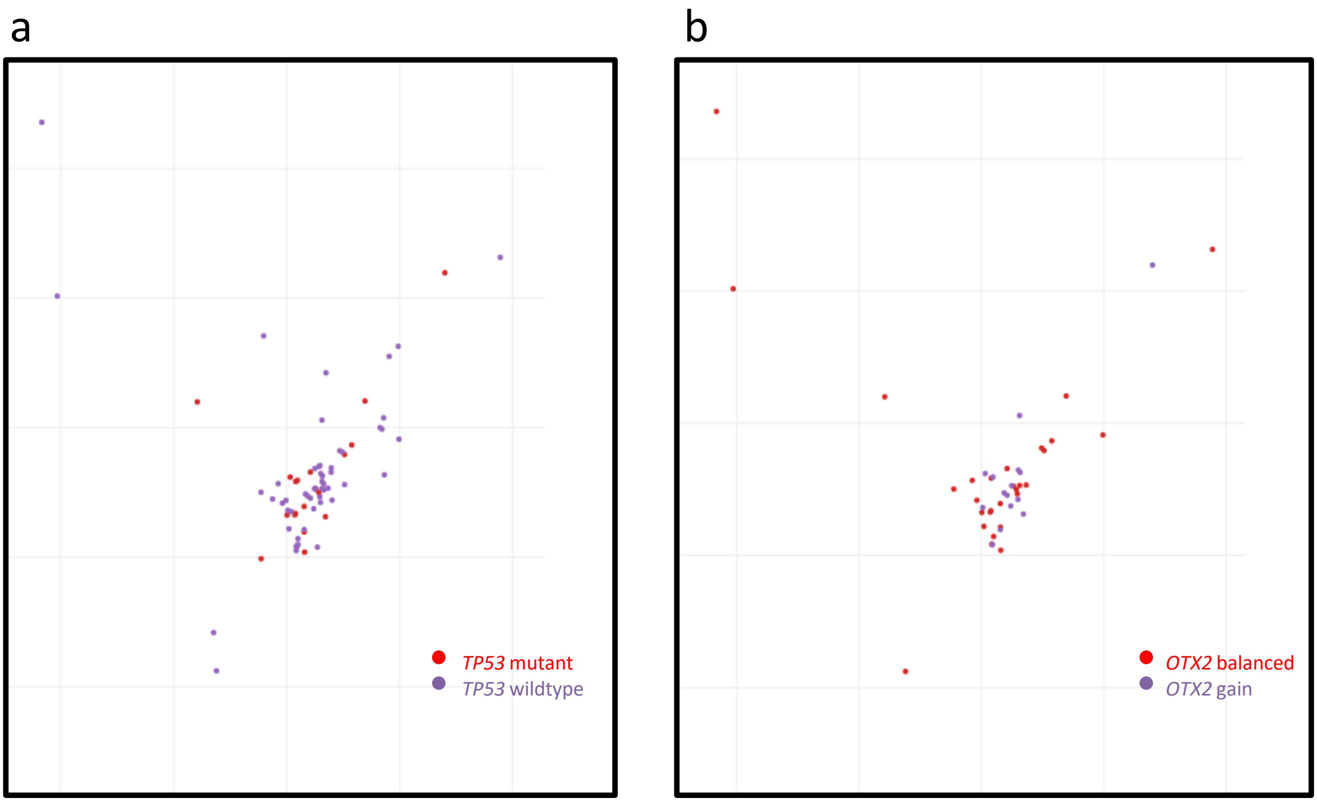

Online Resource Fig. 4. UMAP plot of methylation data of WNT medulloblastoma cases derived from 450k/850 methylation array data (Illumina).
(a) *TP53* mutant versus wildtype cases do not form specific substructures,
(b) cases with gain of *OTX2* versus cases without do not form specific substructures. Note that for some cases, information on *OTX2* status is not available.
For method, see Zschernack et al., Acta Neuropathol. 2021;141(3):455-466. PMID: 33481105

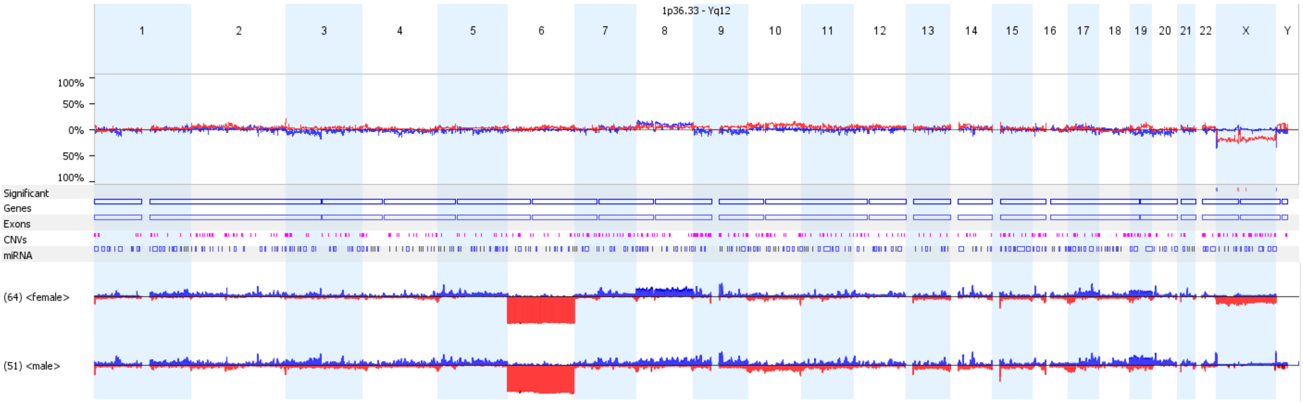

Online Resource Fig. 5: Comparison plot – female versus male

Comparison analysis using Nexus software from female versus male WNT-medulloblastoma samples. Male samples are set as baseline (p = 0.05; differential threshold = 25%). From the 115 samples 108 were analyzed by Molecular Inversion Probe array and 7 by SNP6 array.

CNVs, copy number variations.

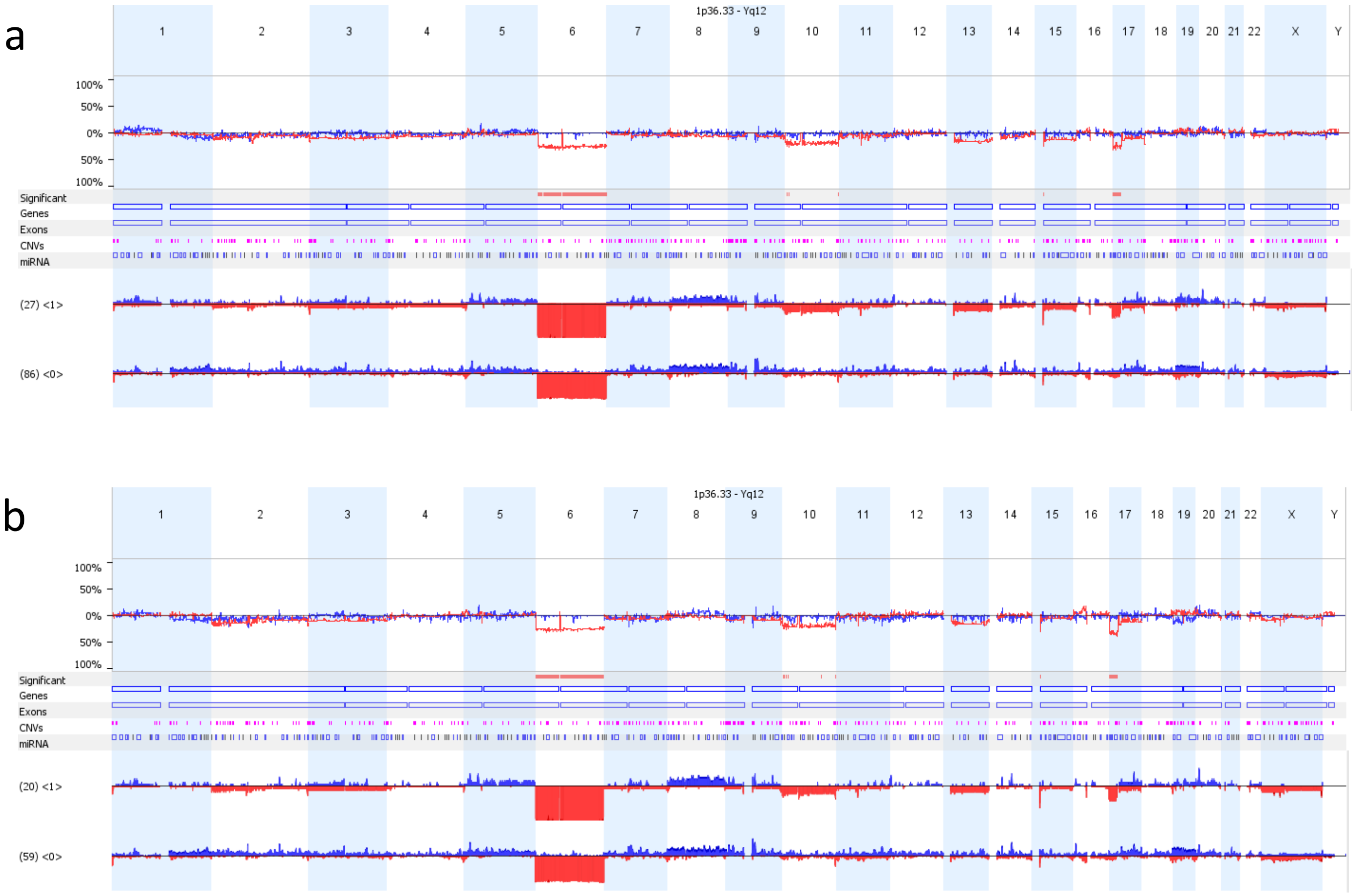

Online Resource Fig. 6: Comparison plots – *TP53* mut. versus *TP53* wt

Comparison analysis using Nexus software from *TP53* mutant (<1>) versus *TP53* wild-type (<0>) WNT-medulloblastoma samples. *TP53* wild-type samples are set as baseline (*P* = .05; differential threshold = 25%). **(a)** All 113 samples with *TP53* sequencing data and analyzed by Molecular Inversion Probe array (n = 106) or SNP6 array (n = 7). **(b)** All 79 samples with clinical data, *TP53* sequencing data, and analyzed by Molecular Inversion Probe array (n = 74) or SNP6 array (n = 5).

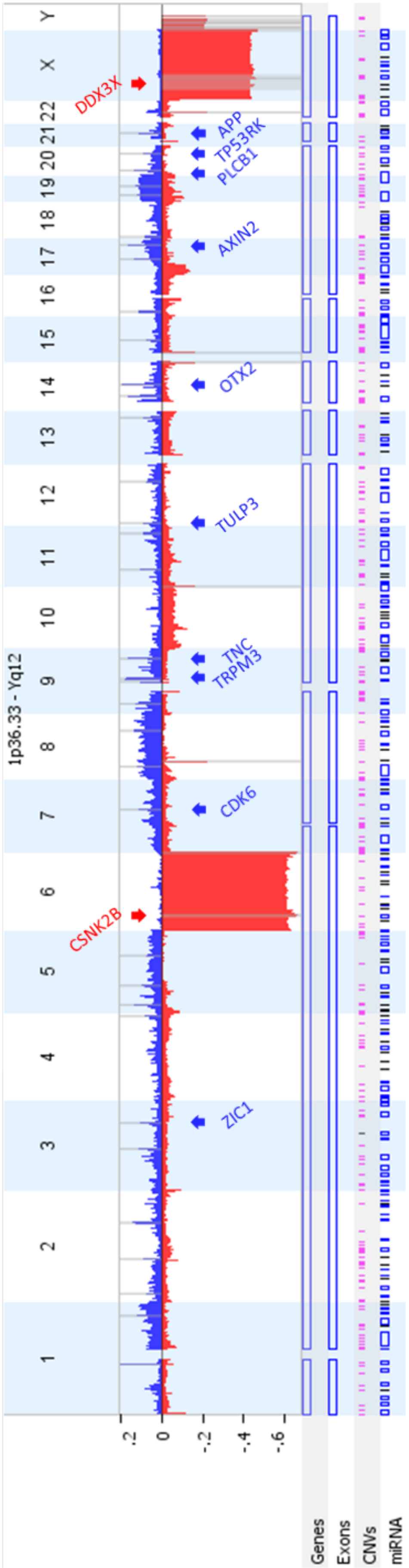

Online Resource Fig. 7 – GISTIC analysis

GISTIC (Genomic Identification of Significant Targets in Cancer) plots (Q-Bound cut-off: 0.005) of WNT-MBs analyzed by Molecular Inversion Probe array (n=108). Detailed table of GISTIC peaks in Online Resource Table 6.

Significant GISTIC regions are shown in darker grey within extended GISTIC regions (light grey). Often GISTIC regions and extended regions are equal (only dark grey).

Gains are shown in blue, losses in red.

CNVs, copy number variations.

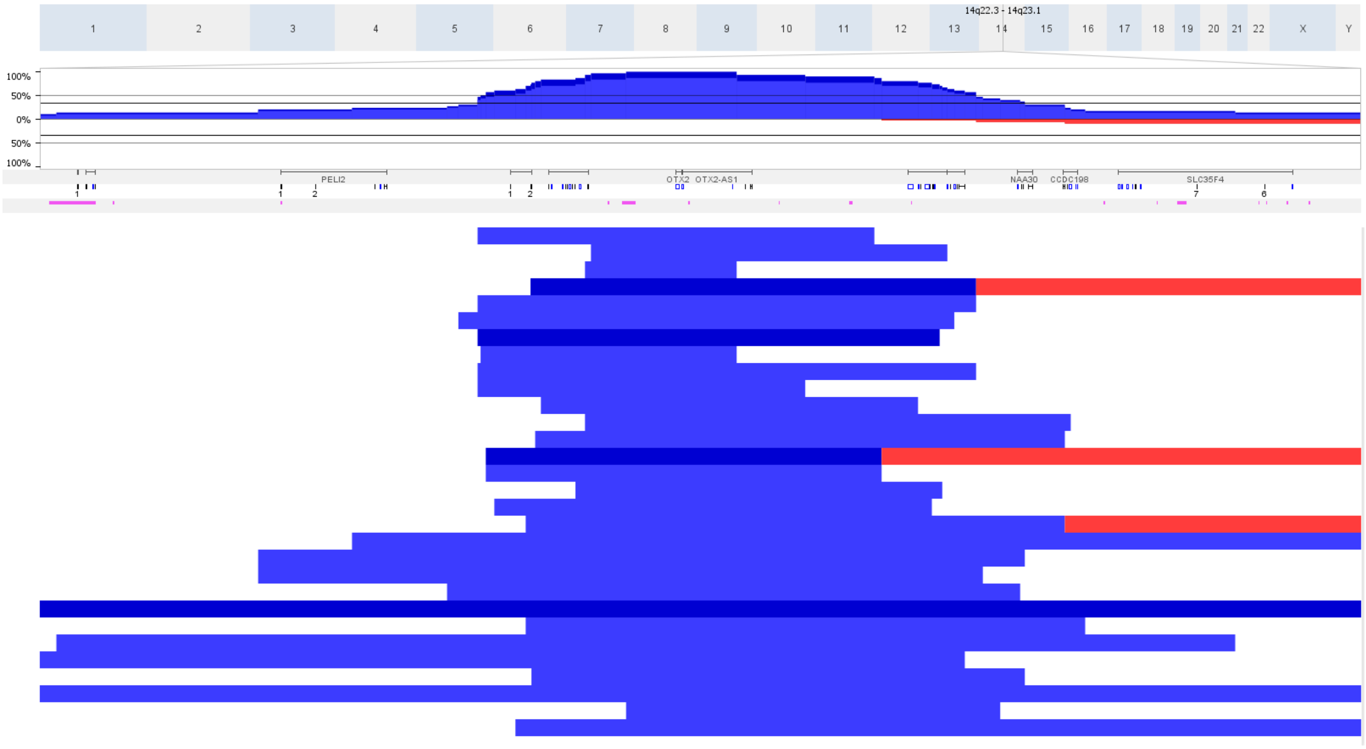

Online Resource Fig. 8: Extent of *OTX2* gains (MIP array).
Enlarged illustration of the *OTX2* region on chromosome arm 14q of all 30 cases with *OTX2* gain and survival data. Of these, 28 have focal gains (blue) or high gains (dark blue), and 2 cases have whole chromosome arm 14q gains.

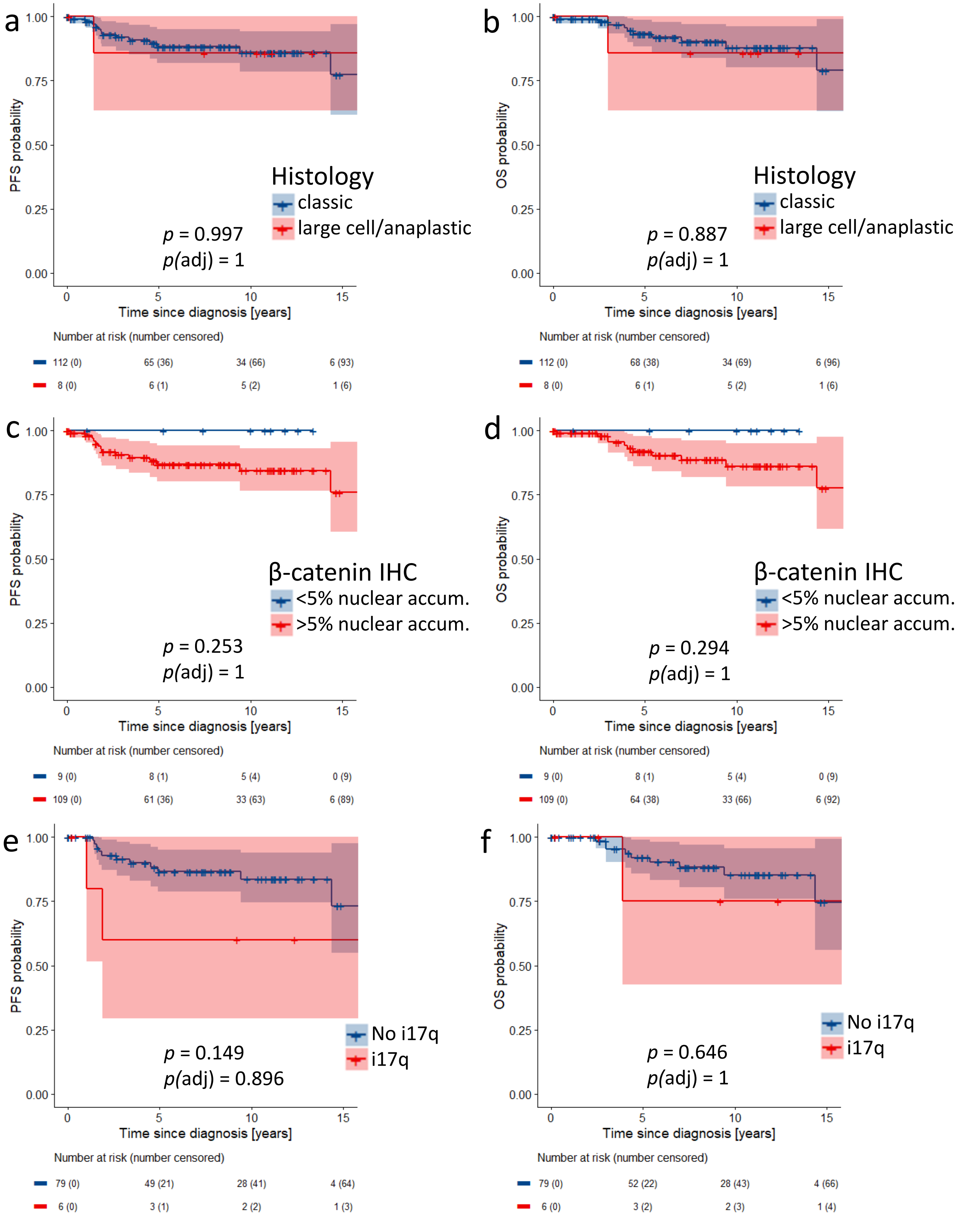

Online Resource Fig. 9: Kaplan–Meier progression-free (PFS) and overall survival (OS) plots for Histology (a, b), β-catenin IHC (c, d), and isochromosome 17q (i17q) versus no i17q (e, f).

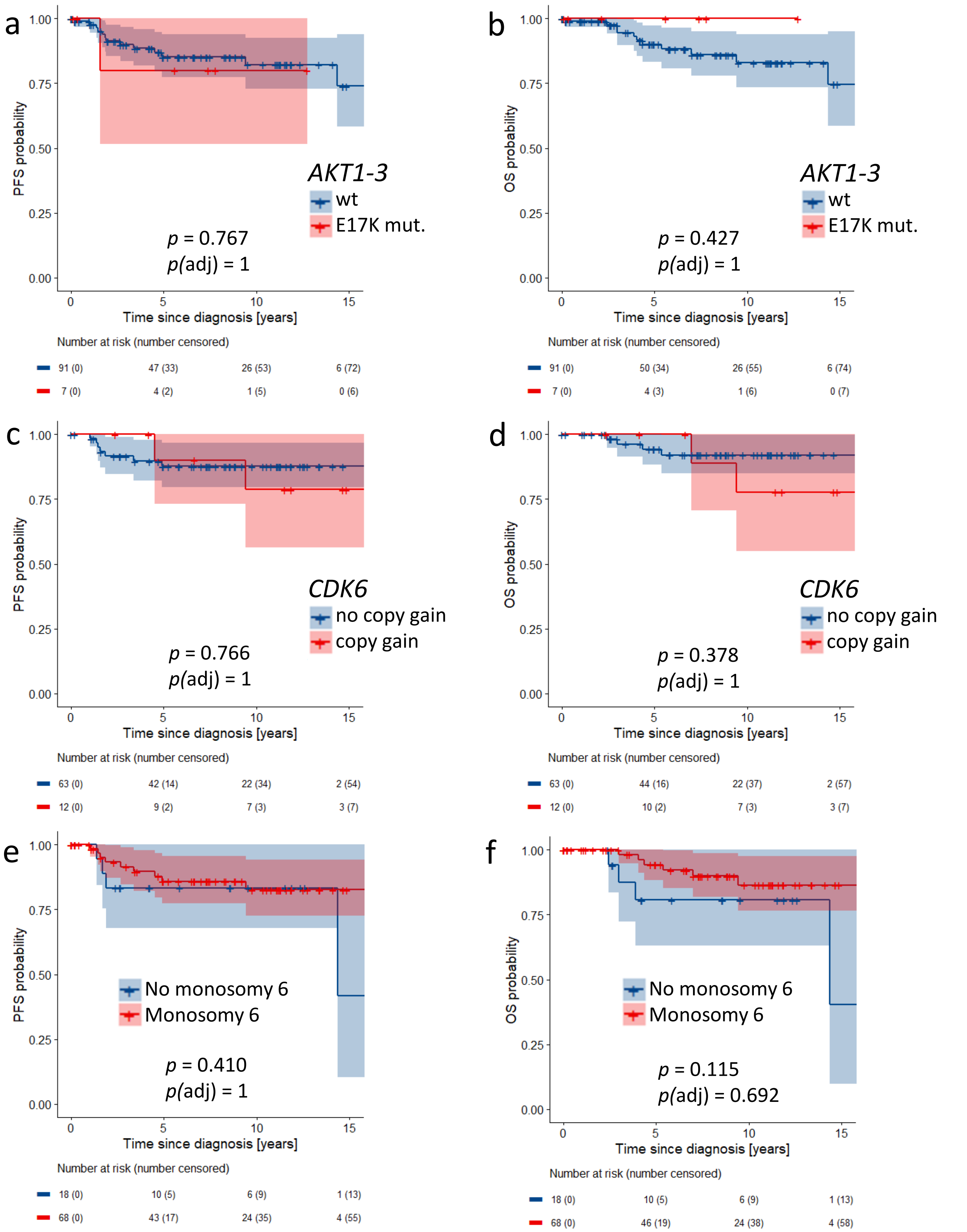

Online Resource Fig. 10: Kaplan–Meier progression-free (PFS) and overall survival (OS) plots for *AKT1-3* mutations (a, b), *CDK6* gain (c, d), and Monosomy 6 (e, f).

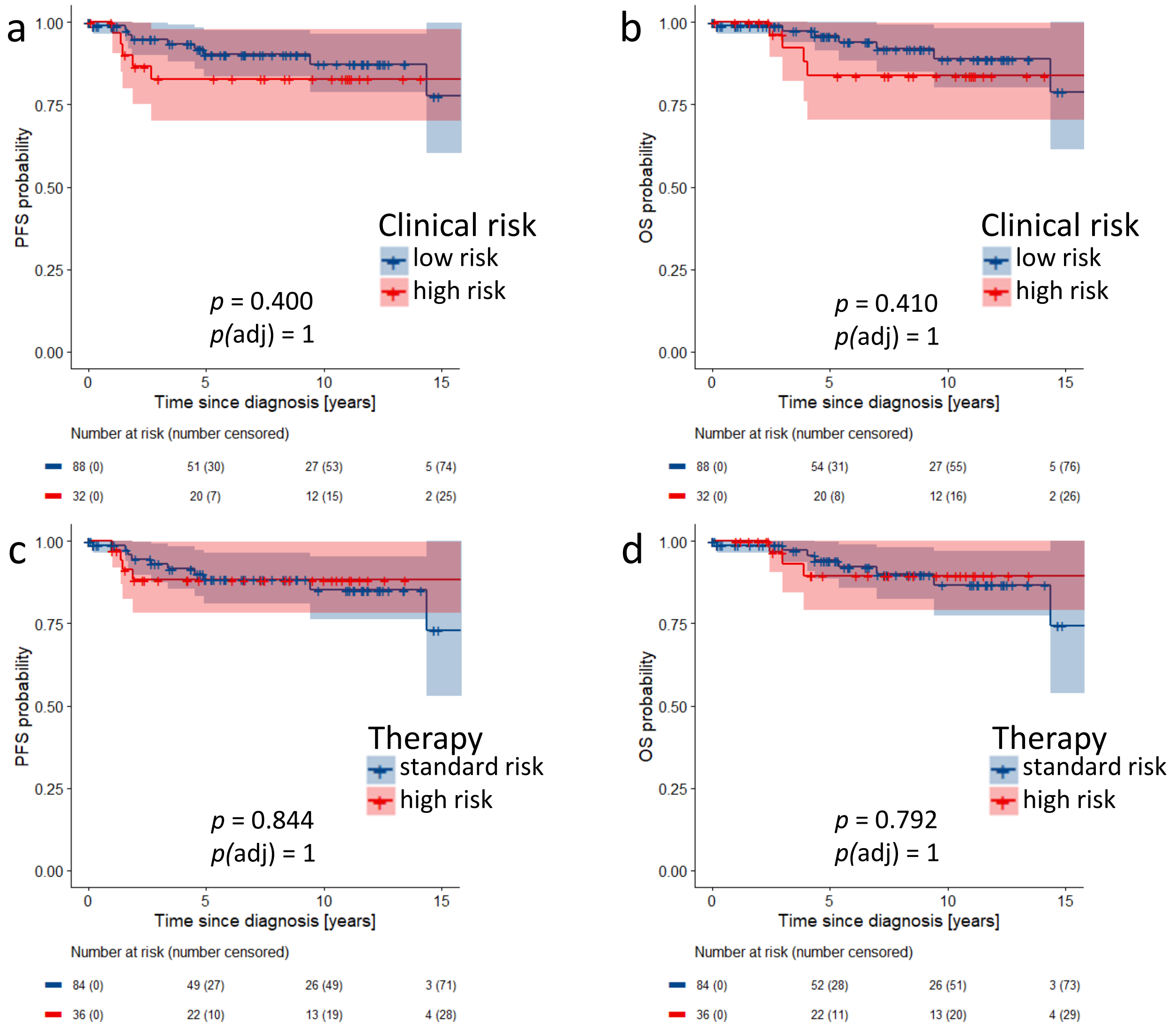

Online Resource Fig. 11: Kaplan–Meier progression-free (PFS) and overall survival (OS) plots for Clinical risk (a, b) and Therapy regimen (c, d).

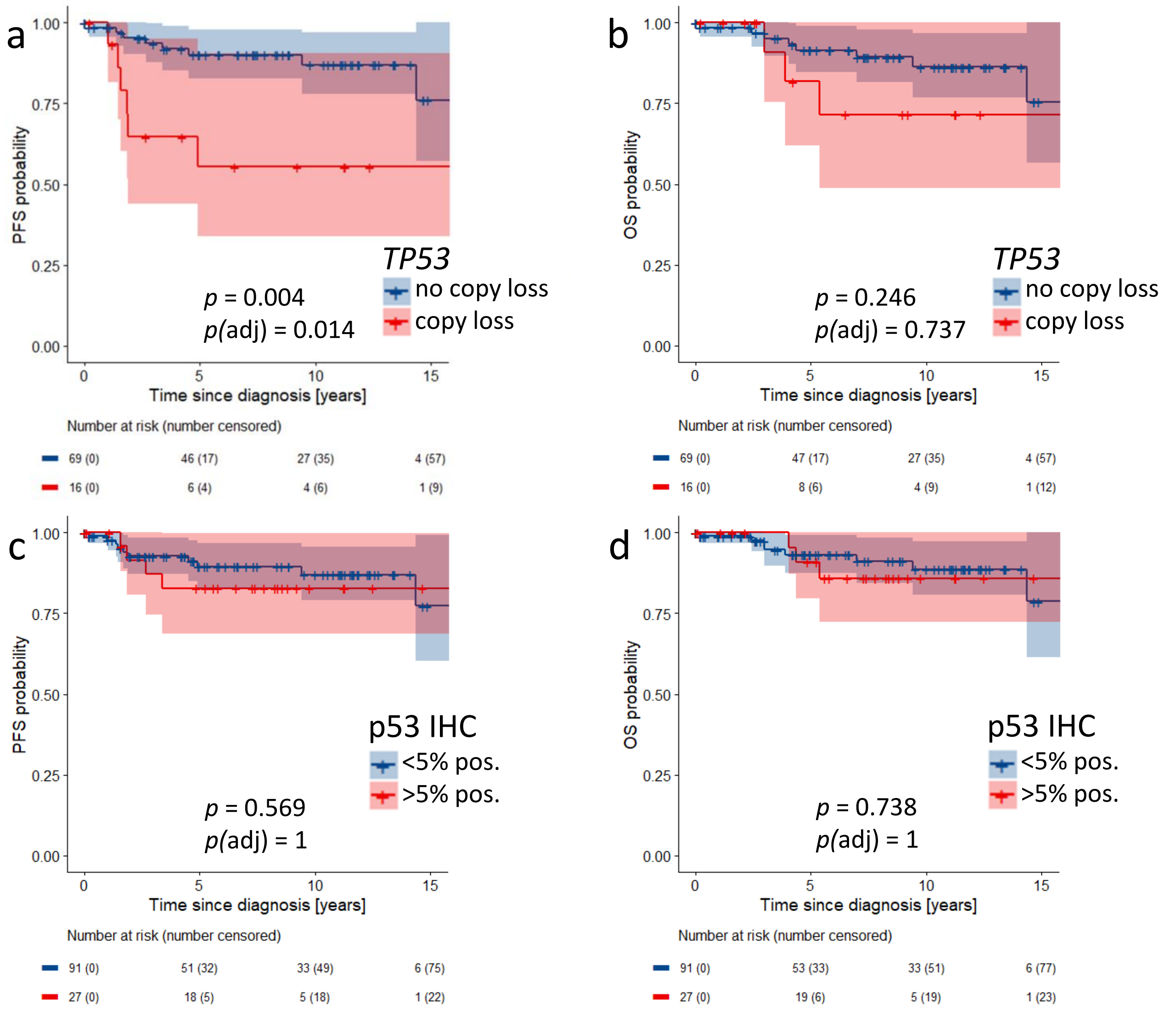

Online Resource Fig. 12: Kaplan–Meier progression-free (PFS) and overall survival (OS) plots for *TP53* loss (a, b) and p53 immunohistochemistry (IHC; c, d).

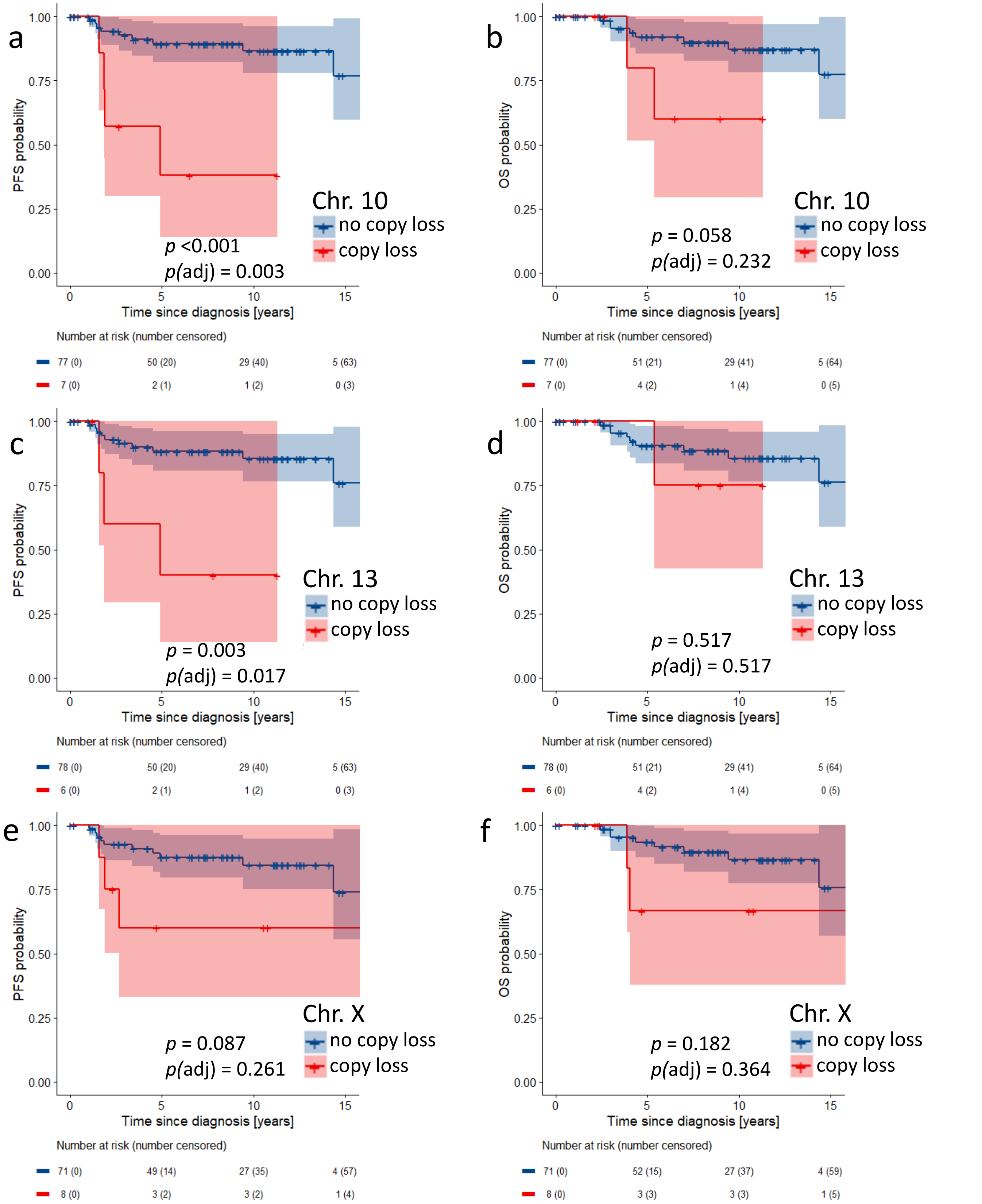

Online Resource Fig. 13: Kaplan–Meier progression-free (PFS) and overall survival (OS) plots for chr. 10 loss (a, b), chr. 13 loss (c, d), and chr. X loss (e, f).

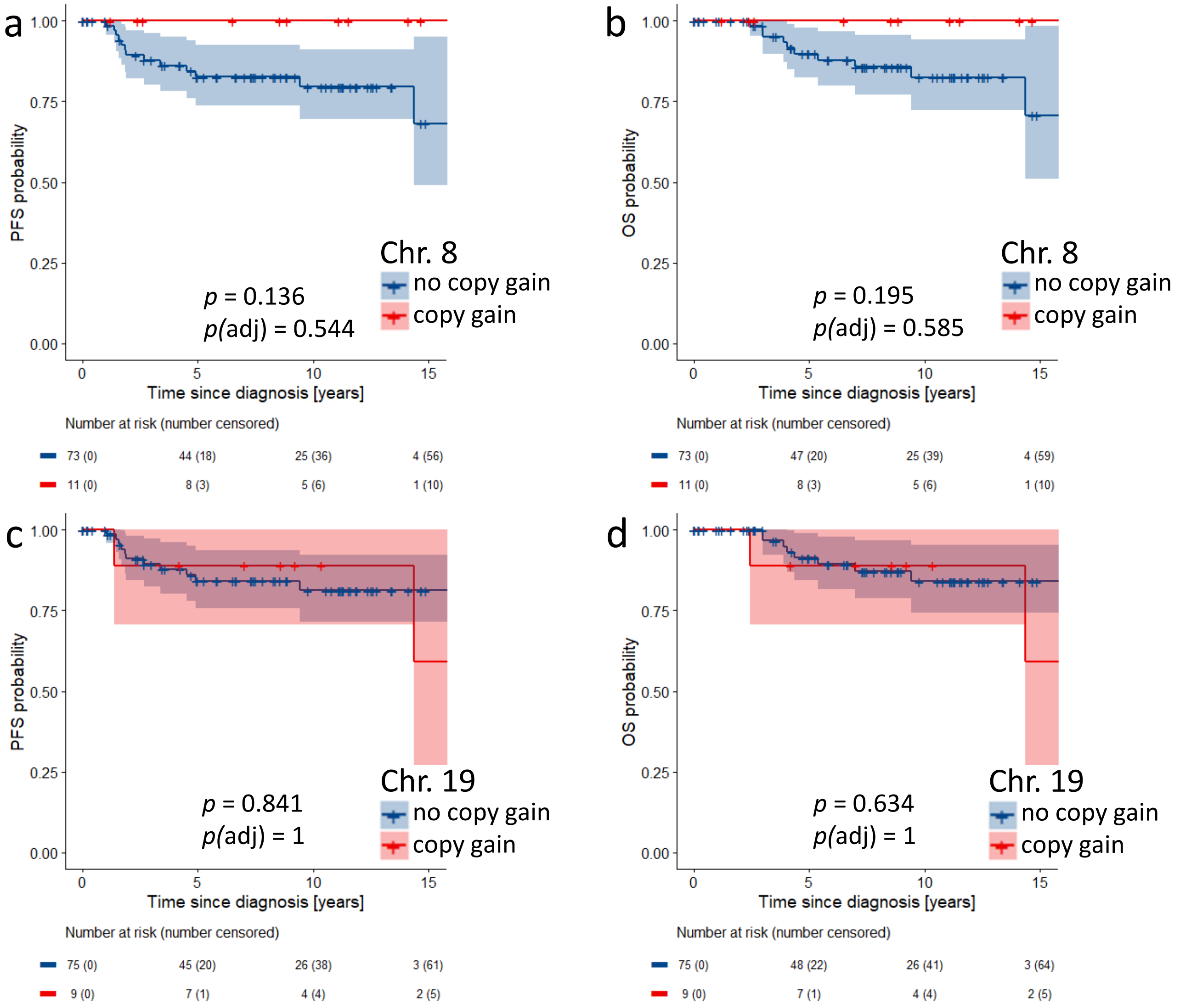

Online Resource Fig. 14: Kaplan–Meier progression-free (PFS) and overall survival (OS) plots for chr. 8 gain (a, b) and chr. 19 gain (c, d).

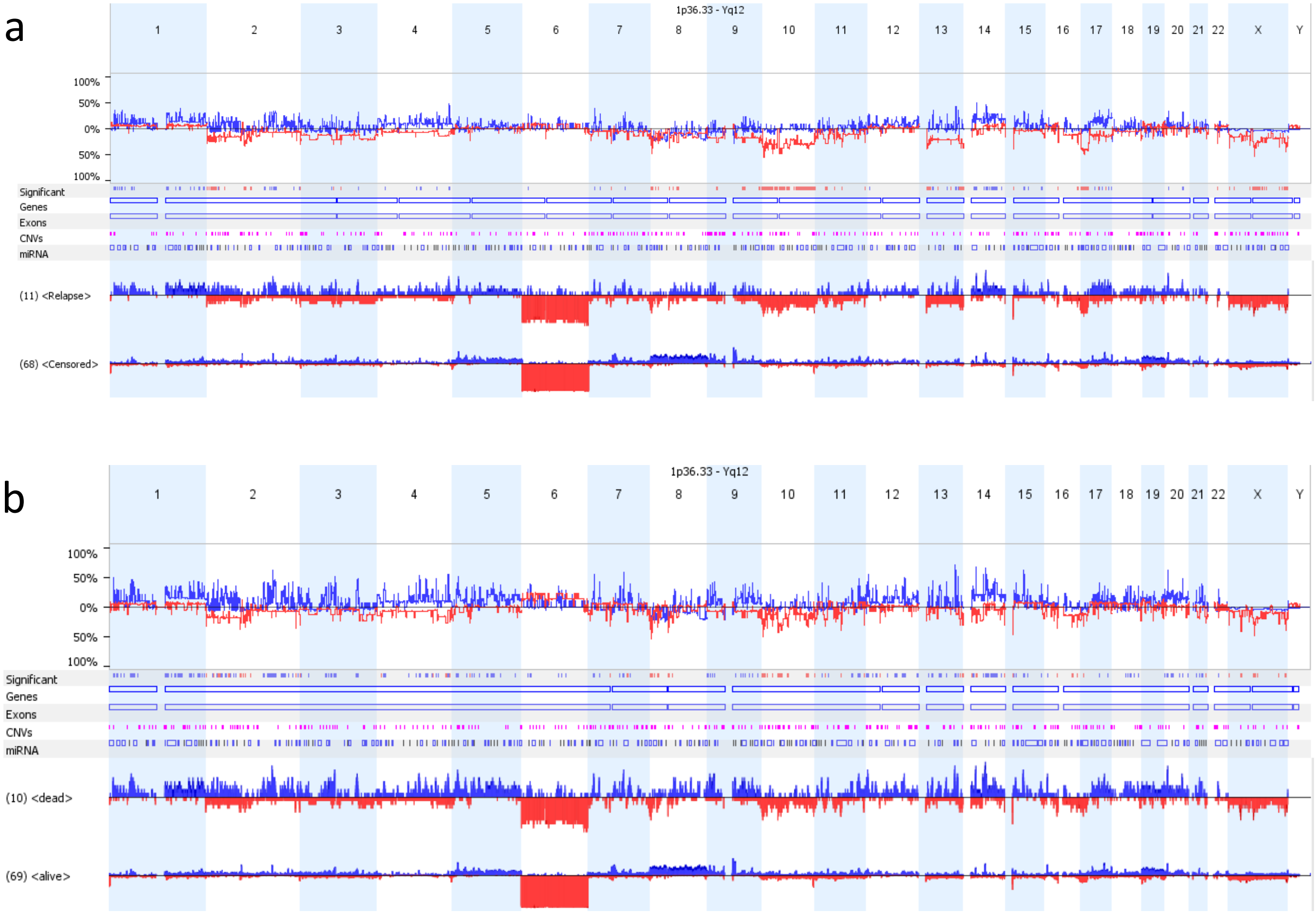

Online Resource Fig. 15: Comparison plots – Relapse and Death

Comparison analysis using Nexus software from WNT-medulloblastoma patients with **(a)** progressive disease or tumor relapse (<Relapse>) versus all others (<Censored>) and **(b)** dead of any reason (<dead>) versus all others (<alive>). Censored **(a)** and alive **(b)** samples are set as baseline (p = 0.05; differential threshold = 25%). (a, b) All 79 samples with clinical data analyzed by Molecular Inversion Probe array (n = 74) or SNP6 array (n = 5).

One patient died from a secondary tumor and one other patient died of unknown reason; all others had tumor relapse and died because of the WNT-medulloblastoma.

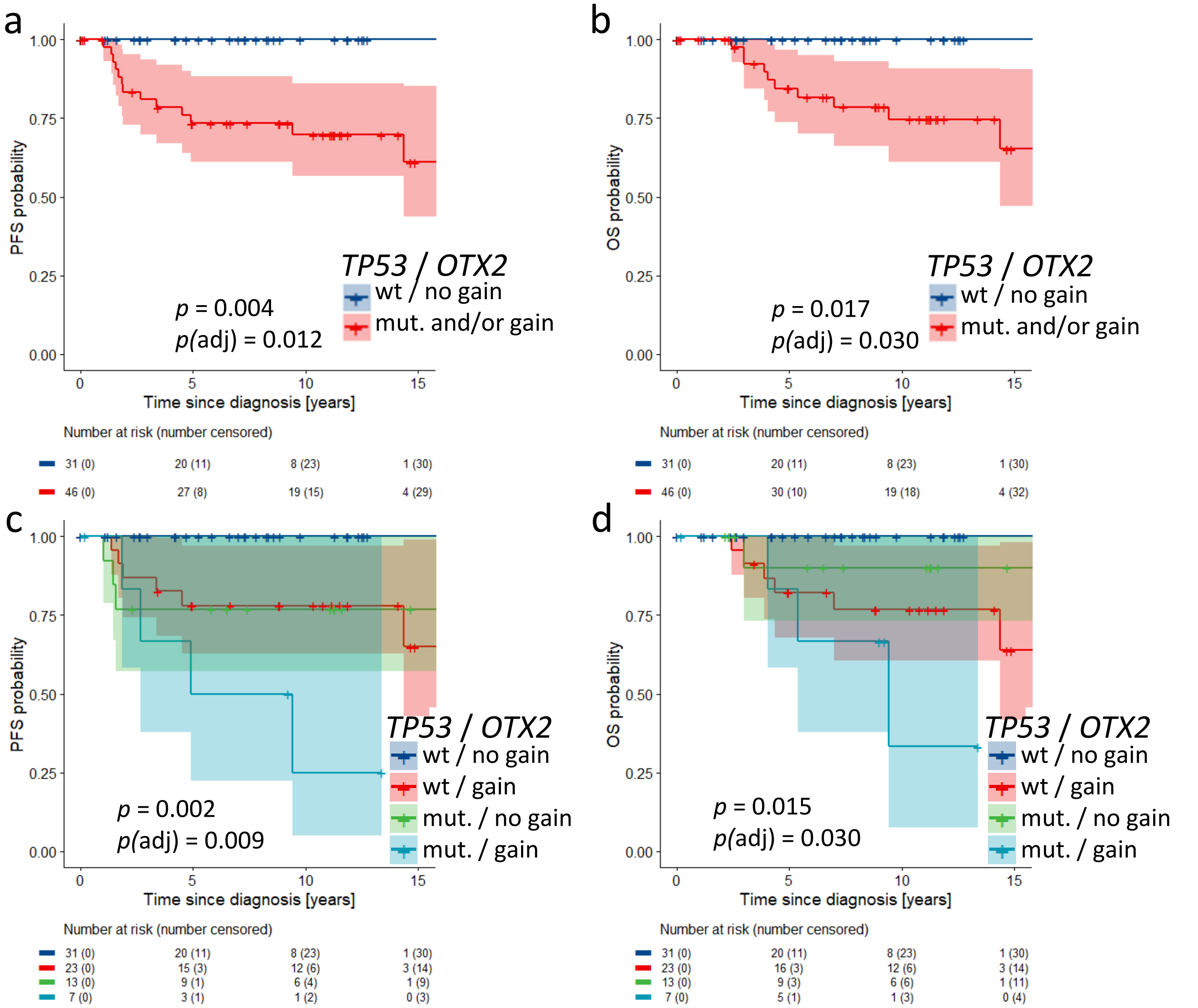

Online Resource Fig. 16: Kaplan–Meier progression-free (PFS) and overall survival (OS) plots for *TP53*wt WNT-MB without *OTX2* gain versus WNT-MB with *TP53* mutation and/or *OTX2* gain (a, b) and WNT-MB according to different combination statuses of *TP53* mut. and/or *OTX2* gain (c, d).

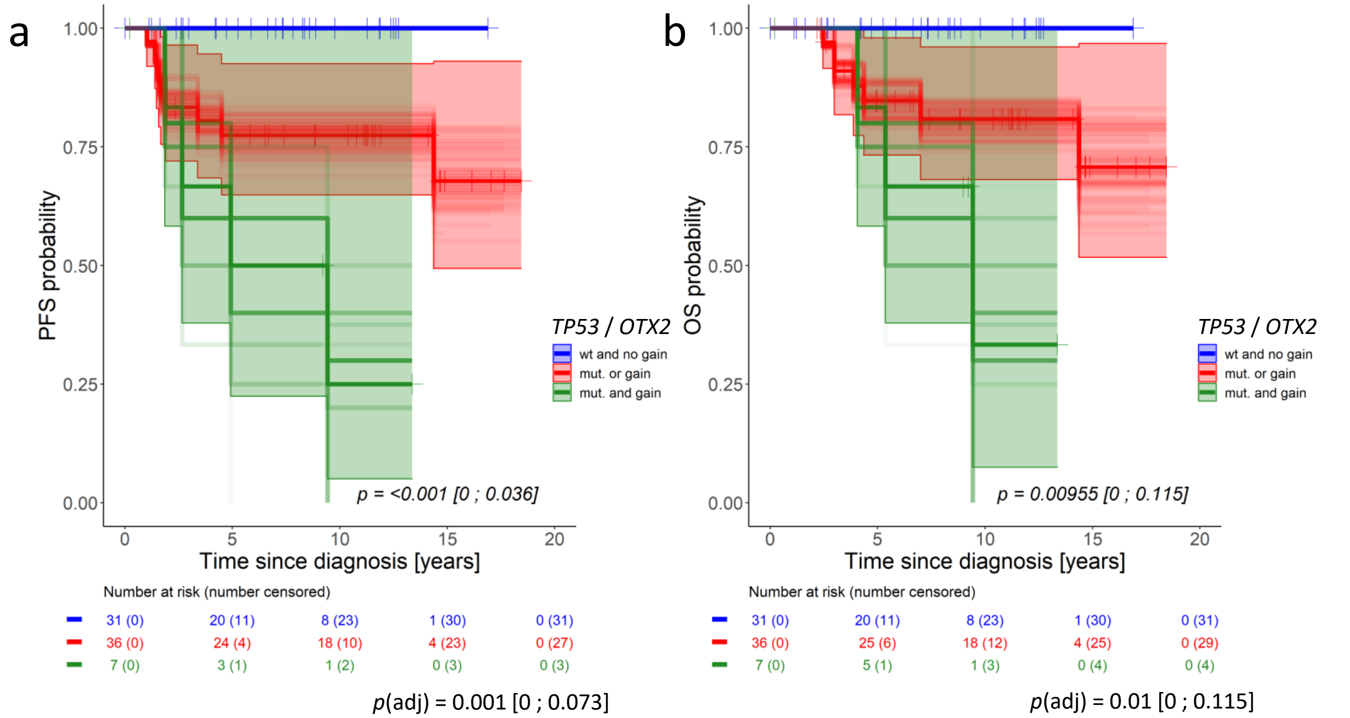

Online Resource Fig. 17: (a, b) Distribution of Kaplan–Meier progression-free (PFS) and overall survival (OS) plots after subsampling of patients with WNT-MBs without *TP53* mutation and *OTX2* gain (blue line), one of these two alterations (red line), or both alterations (green line).

Definition of „pure“ SR sub-cohort by SIOP-PNET5-MB definition (n = 50):

- Age <16
- Non-LCA
- R0/M0
- SR therapy (23.4Gy CSI + Boost + Maintenance)

**Characteristic**	**n = 50**[1]
Age	9.00 (7.25, 12.75)
Sex	
Male	22 (44%)
Female	28 (56%)
Histology	
CLA	50 (100%)
Staging	
M0R0 (<1.5cm2)	50 (100%)
TP53 mutation status	
nd	1 (2.0%)
*TP53*wt	39 (78%)
*TP53*mut	10 (20%)
Activating mutation	
CTNNB1	48 (96%)
APC	2 (4.0%)
OTX2 status	
Nd	19 (38%)
no *OTX2* gain	19 (38%)
OTX2 gain	12 (24%)
Chr 6 status	
nd	14 (28%)
Chr 6 balanced	3 (6.0%)
Monosomy 6	33 (66%)
Clinical Risk Classification	
LR	50 (100%)
Therapy	
SR (23.4Gy CSI + Maintenance)	50 (100%)

[1] **Median (IQR); n (%)**

Kaplan-Meier plots on next page

TP53

a

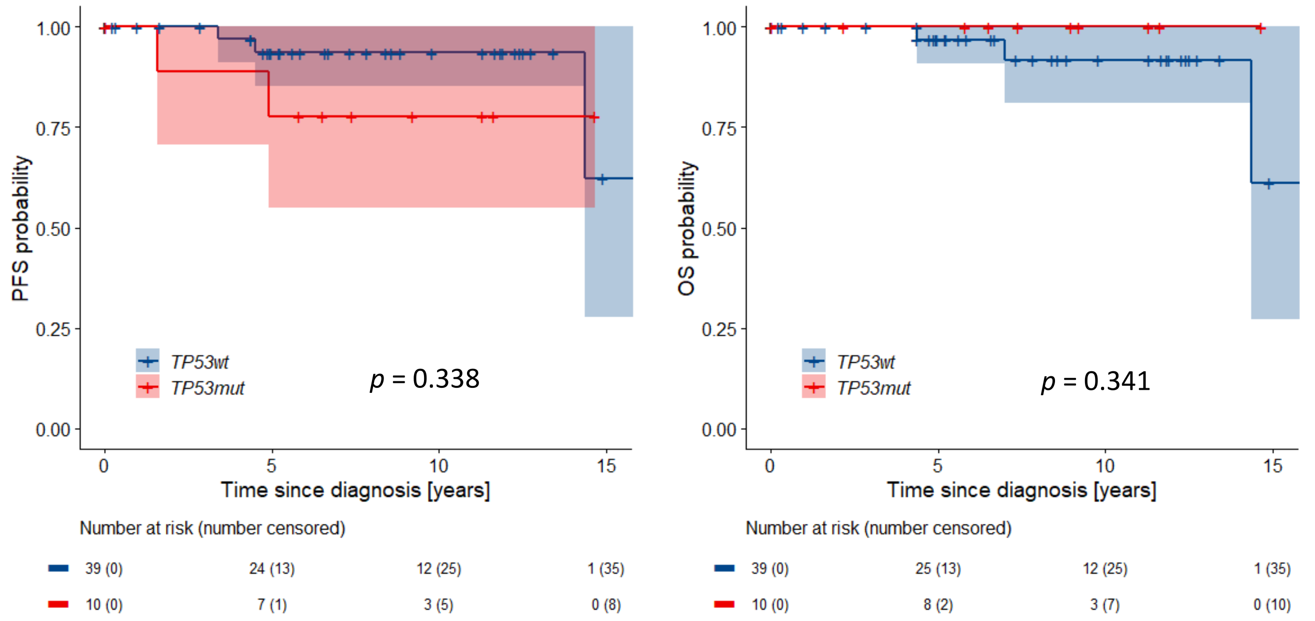

OTX2

b

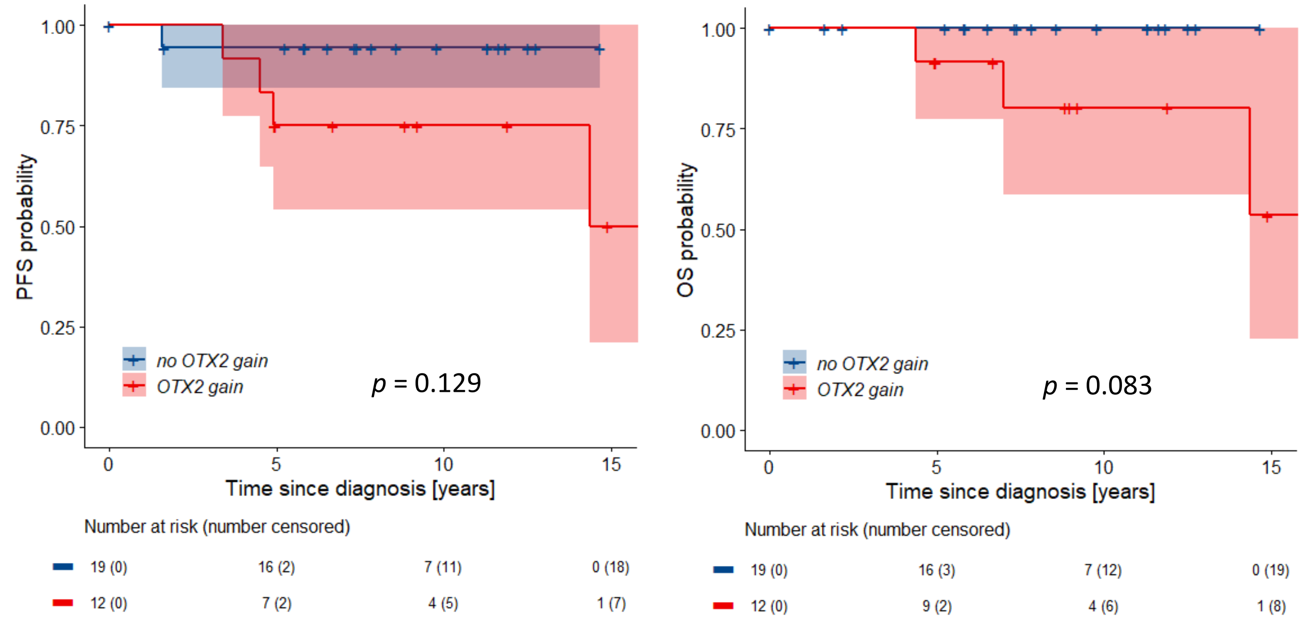

Online Resource Fig. 18: Kaplan–Meier progression-free (PFS) and overall survival (OS) plots for *TP53* mutation (a) and *OTX2* gain (b) from a “pure” standard-risk (SR) cohort (age <16 years).

Definition of „pure“ SR sub-cohort by SJMB12/ACNS1422 definition (n = 64):

- Age <22
- Non-LCA
- R0/M0
- SR therapy (23.4Gy CSI + Boost + Maintenance)

**Characteristic**	**n = 64**[1]
**Age**	11.0 (8.0, 15.0)
**Sex**	
male	27 (42%)
female	37 (58%)
**Histology**	
CLA	64 (100%)
**Staging**	
M0R0 (<1.5cm2)	64 (100%)
***TP53* mutation status**	
nd	1 (1.6%)
*TP53*wt	52 (81%)
*TP53*mut	11 (17%)
**activating mutation**	
CTNNB1 and *APC* wt	1 (1.6%)
CTNNB1	59 (92%)
APC	4 (6.2%)
***OTX2* status**	
nd	25 (39%)
no *OTX2* gain	25 (39%)
OTX2 gain	14 (22%)
**Chr 6 status**	
nd	18 (28%)
Chr 6 balanced	7 (11%)
Monosomy 6	39 (61%)
**Clinical risk classification**	
LR	64 (100%)
**Therapy**	
SR (23.4Gy CSI + Maintenance)	64 (100%)

[1] **Median (IQR); n (%)**

Kaplan-Meier plots on next page

TP53

c

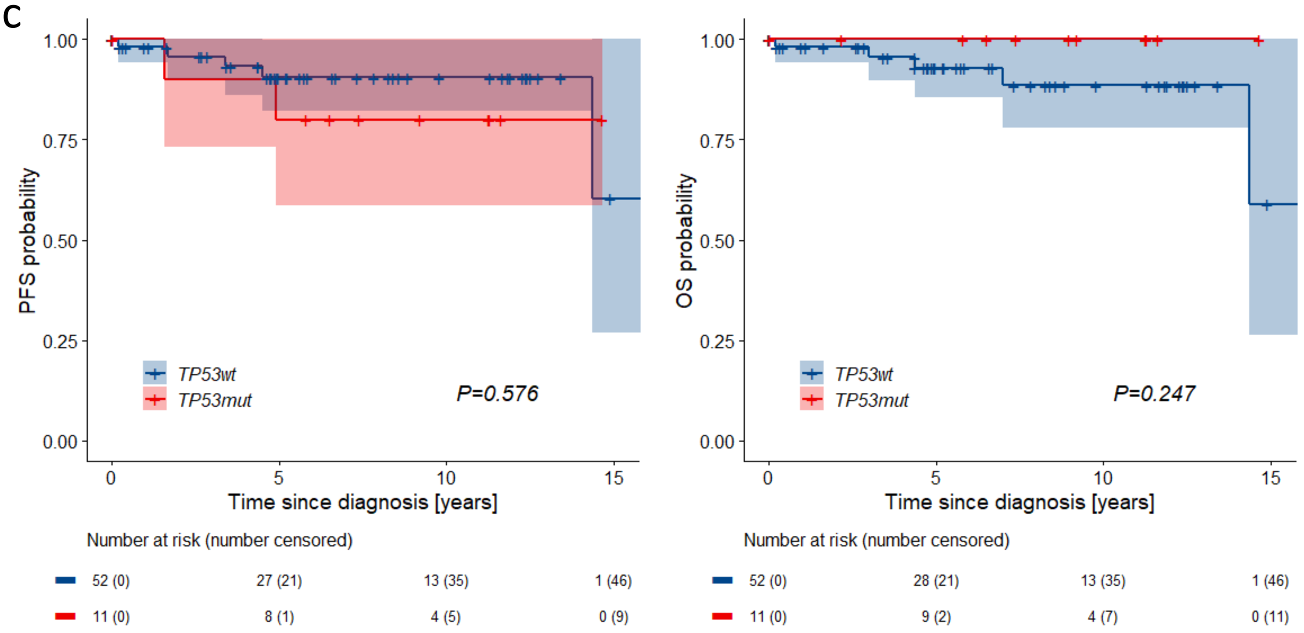

OTX2

d

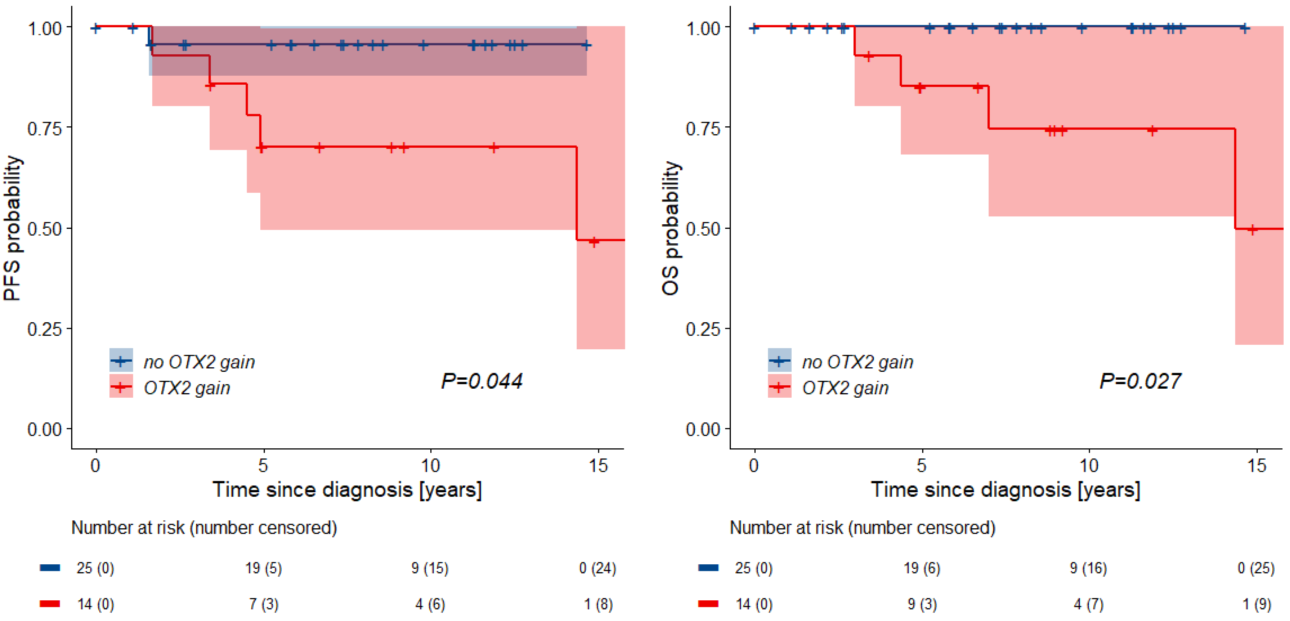

Online Resource Fig. 18: Kaplan–Meier progression-free (PFS) and overall survival (OS) plots for *TP53* mutation (c) and *OTX2* gain (d) from a "pure" standard-risk (SR) cohort (age <22 years).
